# Supplementary material for: Risk of autism spectrum disorder in offspring following paternal use of selective serotonin reuptake inhibitors before conception: a population-based cohort study
Source: BMJ Open. 2017 Dec 22;7(12):e016368. doi: 10.1136/bmjopen-2017-016368 (PMC5770959; doi:10.1136/bmjopen-2017-016368)
Supplement: Supplementary file 1 [file bmjopen-2017-016368supp001.pdf]

TablesS1. Association between paternal SSRIs use before conception and ASD in offspring: analyses stratified by gender

Paternal SSRIs use before conception	Offspring with ASD no.	Follow-up no. of person-yr	Hazard Ratio (95% CI)		
			Crude	Model 1[a]	Model 2[b]
**Boys**					
No use during the last 3 months prior to conception	6,005	3,459,592	Ref	Ref	Ref
Use during the last 3 months prior to conception	84	31,642	1.61(1.30-2.00)	1.55(1.25-1.92)	1.44(1.16-1.79)
Sub-analysis: ***Paternal SSRIs use during the last 1 year before conception***					
No use during the last 1 year prior to conception	5,969	3,444,956	Ref	Ref	Ref
Use only from the last 1 year to the last 3 months prior to conception	58	20,031	1.74(1.34-2.25)	1.69(1.30-2.19)	1.58(1.22-2.05)
Use only during the last 3 months prior to conception	18	7,622	1.42(0.89-2.26)	1.39(0.87-2.21)	1.31(0.83-2.09)
Use both before and during the last 3 months prior to conception	44	18,623	1.44 (1.07-1.93)	1.36(1.01-1.84)	1.26(0.94-1.71)
**Girls**					
No use during the last 3 months prior to conception	1,468	3,305,613	Ref	Ref	Ref
Use during the last 3 months prior to conception	20	29,914	1.63(1.05-2.53)	1.54(0.99-2.40)	1.41(0.90-2.20)
Sub-analysis: ***Paternal SSRIs use during the last 1 year before conception***					
No use during the last 1 year prior to conception	1,460	3,291,698	Ref	Ref	Ref
Use only from the last 1 year to the last 3 months prior to conception	13	19,390	1.61(0.93-2.78)	1.53(0.88-2.64)	1.37(0.79-2.38)
Use only during the last 3 months prior to conception	2	7,116	0.67(0.17-2.69)	0.64(0.16-2.55)	0.59(0.15-2.36)
Use both before and during the last 3 months prior to conception	13	75,323	1.84(1.06-3.17)	1.74(1.01-3.01)	1.59(0.92-2.75)

Abbreviations: AD, antidepressant drugs; SSRI, selective serotonin reuptake inhibitor; ASD, Autism Spectrum Disorder; No., number; HR, Hazard Ratio

[a] Adjusted for calendar year of birth, parity, mother age, father age, maternal smoking, mother psychiatric history, maternal AD use during pregnancy

[b] Model 1 further adjusted for father psychiatric history

TableS2. Association between paternal SSRIs use before conception and ASD in offspring born to mother who neither received antidepressants medication during pregnancy nor had affective disorders before birth of child

Paternal SSRIs use before conception	Offspring with ASD no.	Follow-up no. of person-yr	Hazard Ratio (95% CI)		
			Crude	Model 1[a]	Model 2[b]
No use during the last 3 months prior to conception	7,184	6,627,720	Ref	Ref	Ref
Use during the last 3 months prior to conception	91	57,081	1.55(1.26-1.91)	1.57(1.28-1.93)	1.45(1.18-1.79)
Sub-analysis:					
Paternal SSRIs use during the last 1 year before conception					
No use during the last 1 year prior to conception	7,144	6,600,852	Ref	Ref	Ref
Use only from the last 1 year to the last 3 months prior to conception	64	37,032	1.67(1.30-2.13)	1.68(1.31-2.15)	1.56(1.22-1.99)
Use only during the last 3 months prior to conception	16	13,634	1.13(0.69-1.85)	1.15(0.71-1.89)	1.08(0.66-1.77)
Use both before and during the last 3 months prior to conception	51	33,283	1.50(1.13-1.97)	1.50(1.13-1.97)	1.38(1.05-1.82)

Abbreviations: AD, antidepressant drugs; SSRI, selective serotonin reuptake inhibitor; ASD, Autism Spectrum Disorder; No., number; HR, Hazard Ratio

[a] Adjusted for calendar year of birth, parity, mother age, father age, maternal smoking, mother psychiatric history

[b] Model 1 further adjusted for father psychiatric history
