# Supplementary material for: Risk of autism spectrum disorder in offspring following paternal use of selective serotonin reuptake inhibitors before conception: a population-based cohort study
Source: BMJ Open. 2017 Dec 22;7(12):e016368. doi: 10.1136/bmjopen-2017-016368 (PMC5770959; doi:10.1136/bmjopen-2017-016368)
Supplement: Author's manuscript [file bmjopen-2017-016368.draft_revisions.pdf]

BMJ Open is committed to open peer review. As part of this commitment we make the peer review history of every article we publish publicly available.

When an article is published we post the peer reviewers' comments and the authors' responses online. We also post the versions of the paper that were used during peer review. These are the versions that the peer review comments apply to.

The versions of the paper that follow are the versions that were submitted during the peer review process. They are not the versions of record or the final published versions. They should not be cited or distributed as the published version of this manuscript.

BMJ Open is an open access journal and the full, final, typeset and author-corrected version of record of the manuscript is available on our site with no access controls, subscription charges or pay-per-view fees (http://bmjopen.bmj.com).

If you have any questions on BMJ Open's open peer review process please email editorial.bmjopen@bmj.com

Risk of autism spectrum disorder in offspring following paternal use of selective serotonin reuptake inhibitors before conception

Journal:	*BMJ Open*
Manuscript ID	bmjopen-2017-016368
Article Type:	Research
Date Submitted by the Author:	10-Feb-2017
Complete List of Authors:	Yang, Fen<br>Chen, Jianping; Shanghai Institute of Planned Parenthood Research, Department of Reproductive Epidemiology and Social Science<br>Miao, Mao-Hua; Shanghai Institute of Planned Parenthood Research, Department of Reproductive Epidemiology and Social Science, National Population and Family Planning Key Laboratory of Contraceptive Drugs and Devices<br>Yuan, Wei; Shanghai Institute of Planned Parenthood Research, Reproductive Epidemiology, National Population and Family Planning Key Laboratory of Contraceptive Drugs and Devices;<br>Li, Lin<br>Liang, Hong; Shanghai Institute of Planned Parenthood Research, Department of Reproductive Epidemiology and Social Science, National Population and Family Planning Key Laboratory of Contraceptive Drugs and Devices<br>Ehrenstein, Vera<br>Li, Jiong
**Primary Subject Heading**:	Public health
Secondary Subject Heading:	Mental health
Keywords:	MENTAL HEALTH, Child & adolescent psychiatry < PSYCHIATRY, Adult psychiatry < PSYCHIATRY, NEUROPATHOLOGY

SCHOLARONE™ Manuscripts

Risk of autism spectrum disorder in offspring following paternal use of selective serotonin reuptake inhibitors before conception

Fen Yang[1,2]; Jianping Chen[1]; Maohua Miao[1]; Wei Yuan [1]; Lin Li [1]; Hong Liang[1]; Vera Ehrenstein[2]; Jiong Li [2]

Author Affiliation

[1]Key Lab. of Reproduction Regulation of NPFPC, SIPPR, IRD, Fudan University, Shanghai, 200032, China

[2]Department of Clinical Epidemiology, Aarhus University Hospital, Olof Palmes Alle 43-45, DK-8200 Aarhus N, Denmark

Corresponding Author

Hong Liang, MD, PhD

Key Lab. of Reproduction Regulation of NPFPC, SIPPR, IRD, Fudan University, #779 Lao Hu Min Road, Shanghai, 200032, China.

Email: lucylhcn@163.com;

Phone number: 86 -21-64771683

For peer review only

For peer review only - http://bmjopen.bmj.com/site/about/guidelines.xhtml

Abstract

Objective

The present study aimed to examine the association between paternal selective serotonin reuptake inhibitors (SSRIs) use before conception and the risk of autism spectrum disorder (ASD) in offspring.

Design

A population-based cohort study

Methods

Based on Danish national registers, we conducted a cohort study of 669,922 children born from 1998 to 2008, with follow-up throughout 2013. The children whose fathers used SSRIs during the last 3 months prior to conception were identified as the exposed. Cox regression model was used to estimate the hazard ratio (HR) for ASD in children.

Results

Compared with unexposed children, the exposed had a 1.43-fold higher risk of ASD (HR=1.43, 95% confidence interval [CI]: 1.18-1.74) after adjusting for potential confounders. When extending the exposure window to 1 year before conception, the adjusted HR (aHR) of ASD in children of fathers who used SSRIs only from the last year till the last 3 months prior to conception and who used SSRIs both before and during the last 3 months prior to conception was 1.54 (95%CI: 1.21-1.94) and 1.32 (95%CI: 1.02-1.72), respectively. Whereas, the risk in children of fathers who used SRRIs only during the last 3 months prior to conception decreased and became

For peer review only

nonsignificant (aHR=1.17, 95%CI: 0.75-1.82).

Conclusions

Our findings suggested no substantial increase in the risk of ASD in the offspring attributable to paternal SSRIs use before conception.

Strengths and limitations of this study

- This is the very first study that investigates the link between paternal antidepressants use and ASD risk in children.
- A number of potential confounders, including sociodemographic factors as well as parental psychiatric history, can be adjusted for based on the availability of national health registry data.
- Actual use of SSRIs by fathers during the time period of interest may not be validated.
- Those children who received the diagnosis of ASD from private psychiatrists or remained undiagnosed at the end of the follow-up may not be identified as ASD

For peer review only - http://bmjopen.bmj.com/site/about/guidelines.xhtml

Introduction

Depression is a common mental health problem that affects approximately 350 million people worldwide[1]. Selective serotonin reuptake inhibitors (SSRIs) are commonly prescribed for the treatment of depression and other anxiety-related disorders. However, an increasing body of evidence suggests that prenatal exposure to SSRIs is associated with adverse obstetrical and neonatal outcomes [2-4]. In addition, recent studies have indicated a possible link between prenatal SSRIs exposure and neurobehavioral problems in children[5, 6].

Autism spectrum disorder (ASD) is one of the major neurodevelopmental disorders characterized by impairments in reciprocal social interaction, communication, and repetitive or stereotypic behavior[7]. The estimated prevalence of ASD has dramatically increased from less than 1 in 2,000 children in 1960s to approximately 1 in 145 children today[8, 9]. Both genetic and environmental risk factors may contribute to ASD [10]. Recently, maternal use of SSRIs during pregnancy has been reported to be associated with an increased risk of ASD in children [11-13].

If maternal SSRIs exposure during pregnancy plays a role in fetal, even in child's, development, an extended effect of earlier exposure on gamete, particularly human sperm, is biologically plausible. Therefore, the potential role of paternal SSRIs use in ASD should also be taken into consideration. Studies in the Nordic countries show that one-third of the fathers used prescription drugs during the last 6 months prior to conception [14] , and approximately 1.4% of fathers were dispensed SSRIs during the last 3 months prior to conception [15, 16]. Although the information regarding the

For peer review only

potential effects of SSRI use by fathers is limited, the hypothesis of an increased risk of adverse pregnancy or neonatal outcome associated with paternal drug exposure is not new. Evidence from experimental studies suggests that paternal exposure to a broad range of xenobiotic agents may induce reproductive and developmental abnormalities in the subsequent offspring, e.g., spontaneous abortions, congenital malformations, growth retardation, neurobehavioral deficits, or carcinogenesis[17-19]. Epidemiologic studies have also shown that certain pharmacological agents used by fathers before conception may increase the risk of spontaneous abortions, birth defects or childhood cancers[20, 21]. The potential mechanisms behind the male-mediated effects include: 1) genetic or epigenetic changes with direct disturbances in spermatocytes; 2) indirect effects by transmission of the xenobiotic agents to the female via the seminal fluid[17, 18]. Several studies have indicated the adverse effects of paternal SSRIs use, including impaired semen quality and abnormal sperm DNA fragmentation [22, 23], which has been reported to be associated with diminished fertility, adverse pregnancy outcomes, and an increased risk of childhood disease [24].

Little is known on whether paternal SSRIs use before conception contributes to the risk of ASD in offspring. We conducted a population-based cohort study to examine the association between paternal SSRIs use during the last 3 months prior to conception and risk of ASD in offspring, utilizing data from national Danish health registries.

Methods

For peer review only - http://bmjopen.bmj.com/site/about/guidelines.xhtml

Study Population

This study was based on several national registers in Denmark. Each Danish resident is assigned a unique personal identification number (a 10-digit civil registration system number used in all registries), which enables accurate linkage of national registries at the individual level[25]. The Danish Medical Birth Registry (DMBR) contains records of all deliveries in Denmark since 1973 and includes information about gestational age at birth from 1978[26].Using the DMBR data, we identified a cohort of all singletons born alive in Denmark during the period of January 1, 1998 through December 31, 2008 (n=687,580). We excluded children without linkage to their father (n=16,955) or their mother (n=74), with missing parity (n=208), and with missing or extreme values of gestational age (≤23 weeks or ≥45 weeks, n=421). A total of 669,922 children were included in the analysis.

Data on SSRIs use

Information on SSRIs use was drawn from the Danish National Prescription Registry (DNPR)[27]. Since 1995, this registry has recorded all redeemed prescriptions in Denmark with the following information: the civil registration number of the patient, the dispensing date, the medication code (the WHO Anatomical Therapeutic Chemical (ATC) classification system), the number of packages prescribed, and the number of doses units in package. Selective serotonin reuptake inhibitors use was identified based on ATC codes: fluoxetine (N06AB03), citalopram (N06AB04), paroxetine (N06AB05), sertraline (N06AB06), fluvoxamine (N06AB08), and escitalopram (N06AB10). It is estimated that spermatogenesis takes approximately 74 days[20],

For peer review only - http://bmjopen.bmj.com/site/about/guidelines.xhtml

therefore we chose the last 3 months prior to conception as the exposure window to cover the susceptible time period. A child was considered exposed if the dispensing date fell within the specified exposure window or the number of days for which the SSRIs medication was supplied overlapped any portion of the exposure window. Children born to fathers who had no prescriptions for SSRIs and no supply overlap during the entire exposure window were considered unexposed. The date of conception was estimated by subtracting gestational age from the date of delivery. Data on paternal SSRIs use during the last two years prior to the conception were extracted for further analyses. We also retrieved the information about maternal SSRIs use during the pregnancy.

Autism spectrum disorders

Autism spectrum disorders in children were identified by using the Danish Psychiatric Central Research Register (DPCRR) and the Danish National Patient Register (DNPR). The DPCRR contains diagnostic information on every admission from psychiatric hospitals and psychiatric wards in general hospitals in Denmark since 1969, and includes data on all outpatient visits and emergency room contacts since 1995[28]. The DNPR has collected data on all inpatients from all somatic hospitals in Denmark since 1977 and outpatients from 1995[29]. The combined data from the two registries were used to identify all children diagnosed with ASD. During the study period, the diagnosis of ASD was based on the International Classification of Diseases, 10th version (ICD-10) codes of F84.0 (Childhood autism), F84.1 (Atypical autism), F84.5 (Asperger syndrome), F84.8 (other pervasive developmental

For peer review only

disorders) and F84.9 (unspecified pervasive developmental disorders). The quality of the childhood autism diagnosis has been validated and the diagnoses could be verified in 94% of the children with a record in the DPCRR[30]. Children were followed up from birth till first diagnosis of ASD, death, emigration, or December 31, 2013, whichever came first.

Covariates

Using the Danish nationwide health registers, we retrieved data on characteristics that may be associated with offspring ASD or paternal SSRIs use. For each child, we obtained information on calendar year of birth, gender of the child, birth weight, Apgar score at 5 minutes, gestational age, maternal parity, maternal age at child birth, paternal age at child birth and maternal smoking status during pregnancy from the DMBR. Parents' psychiatric history prior to birth of the index child was obtained from DPCRR by ICD-8 codes 290-315 from 1977 to 1993 and ICD-10 codes F00-F99 from 1994 onward. Furthermore, we identified parents diagnosed with affective disorders before birth of child (specifically, ICD-8 codes 296.09, 296.19, 296.29, 296.39, 296.99, 298.09, 298.19, 300.49, and 301.19, and ICD-10 codes F30–F34 and F38–F39) using the DPCRR.

Statistical Analysis

Cox proportional-hazards regression models were used to estimate the hazard ratios (HRs) and 95% confidence intervals (CIs) of ASD in children following exposure to paternal SSRIs use before conception.

For peer review only

For peer review only - http://bmjopen.bmj.com/site/about/guidelines.xhtml

We adjusted for variables as following: the calendar year of birth (1998-2000, 2001-2003, 2004-2006, 2007-2008), gender of the child (boy, girl), parity (1, 2, ≥3), parental age at child birth (≤25, 26-30, 31-35, and >35 years), maternal smoking status during pregnancy (yes or no), maternal history of psychiatric disorders before birth of child (yes or no), maternal antidepressants use during pregnancy (yes or no) in model 1. We additionally adjusted for paternal history of psychiatric disorders before birth of child (yes or no) in model 2. Models were also run with the exclusion of those children with missing data for covariates.

To distinguish the direct effects of SSRIs use from the effects of the indication of SSRIs use, we extended the exposure window to 1 year before conception. We then re-categorized the exposed children into three subgroups: children of fathers who used SSRIs 1) only from the last year till the last 3 months prior to conception (former SSRIs users); 2) only during the last 3 months prior to conception (current SSRIs users); 3) both before and during the last 3 months prior to conception (both former and current SSRIs users). The reference group consisted of those children born to fathers who never used SSRIs medication through the last year before conception.

The stratified analysis was performed to examine whether the association between paternal SSRIs use and ASD in children differed by gender. We also restricted the analyses to children whose mothers neither received antidepressant medication during pregnancy nor had affective disorder before birth of child. To further distinguish the effect of SSRIs medication from that of paternal affective disorders, we performed

For peer review only - http://bmjopen.bmj.com/site/about/guidelines.xhtml

stratified analyses according to paternal history of affective disorder before birth of the index child.

Besides, to control for unmeasured family-related confounding factors, we re-conducted a sibling study by restricting to families with more than one child and with at least one child with paternal SSRIs preconception exposure. Using the stratified Cox proportional-hazards regression, we then compared exposed siblings to unexposed siblings to estimate the association between paternal SSRIs use before conception and ASD in children.

All analyses were performed using SAS version 9.1 (SAS Institute Inc, Cary, North Carolina, USA).

Results

Among the 669,922 singletons included in the study, 6,870 (1.03%) children were born to fathers who had redeemed a prescription for SSRIs during the last 3 months prior to conception. During the study period, a total of 7,577 children were diagnosed with ASD. The median age at diagnosis of ASD was 6.77 years (interquartile range: 4.84 to 9.48 years). Characteristics of the study population are shown in Table 1. Compared with the unexposed group, there were a higher proportion of exposed children born in later calendar years. Fathers in exposed group were more likely to be older at child birth and to have a history of psychiatric disorder (including affective disorder) before birth of child. Mothers of exposed children were characterized as having higher parity, being more likely to be older at child birth, to use

For peer review only - http://bmjopen.bmj.com/site/about/guidelines.xhtml

antidepressants during pregnancy, and to have a history of psychiatric disorder before birth of child.

Among 61,555 person-years of follow-up, we identified 104 cases of ASD in children born to fathers who used SSRIs during the last 3 months prior to conception (incidence rate: 169 per 100,000 person-years). This incidence rate corresponded to a 62% increased risk of ASD compared with the unexposed group (Table 2). The adjusted HR (aHR) of ASD was 1.54 (95% CI: 1.27-1.88) after adjusting for potential confounders in model 1. After paternal psychiatric history was further adjusted for in model 2, the estimate was 1.43 (95%CI: 1.18-1.74). When extending the exposure window to the last one year prior to conception, the aHR for ASD in children of fathers who were former users only and who were both former and current users was 1.54 (95%CI: 1.21-1.94) and 1.32 (95%CI: 1.02-1.72), respectively. However, the increased risk decreased and became nonsignificant among children of fathers who were only current users (aHR=1.17, 95%CI: 0.75-1.82, model 2, table2).

The risk estimates of ASD were similar for both boys and girls, regardless of the exposure (Table S1). When we restricted analyses to children whose mothers neither used SSRIs during pregnancy nor had affective disorder before birth of child, the results did not change essentially (Table S2).

When the analyses were stratified by paternal affective disorder before birth of the index child, in children born to fathers with a history of affective disorder, there was no association between paternal SSRIs use before conception and ASD in the offspring (model 2: aHR=1.10, 95%CI: 0.61-1.98). For children whose father without

For peer review only - http://bmjopen.bmj.com/site/about/guidelines.xhtml

affective disorder, the patterns of associations remained similar to those of the main analyses (Table 3).

In the sibling analysis, we identified 5,479 families with more than one child and with at least one child with paternal SSRIs use before conception (Table 4). The risk of ASD in exposed children was similar when compared with their unexposed siblings (aHR=1.04, 95%CI: 0.94-1.14).

Discussion

In this large population-based cohort study, we observed an increased crude risk of ASD in the offspring following paternal use of SSRIs during the last 3 months prior to conception. However, the risk attenuated after adjusting for a number of potential confounders, especially fathers' psychiatric conditions. When extending the exposure window to one year before conception, the ASD risk persisted among children born to former users but not current users. In addition, among children born to father with affective disorder, no association was observed. Finally, we performed a sibling analysis which allowed for better control of unmeasured familial confounding and no difference in the ASD risk was found among exposed children and their unexposed siblings. Taken as a whole, our results did not support that paternal SSRIs use before conception could increase the risk of ASD in children.

Recently, concerns have been raised regarding the risk of ASD in the offspring associated with prenatal exposure to SSRIs. Three previous human studies have suggested that in utero exposure to antidepressants would increase the risk of ASD in children[11-13], although another two studies using the Danish registers have reported no

For peer review only - http://bmjopen.bmj.com/site/about/guidelines.xhtml

significant association [31, 32]. Basic neuro-biologic studies have showed that prenatal SSRIs administration may be part of a causal pathway to ASD by operating directly on the developing brain[33, 34]. Considering the detrimental effect of SSRIs on sperm [22, 23, 35], paternal SSRIs use before conception may also cause adverse effects on pregnancy outcomes. However, till now, only one study have suggested that SSRIs administration to male rats could induce deterioration in fertility and fetal outcomes (including weight gain, organ weights and feed consumption)[36]. To our knowledge, our study is the first to investigate the link between paternal antidepressants use and ASD risk in children. Considering the limited number of ASD cases and the challenges posed by confounding, further studies are needed to corroborate the findings.

In the present study, the increased risk of ASD observed in former users but not in current users implied that indications for paternal SSRIs use may account for the observed association between paternal SSRIs use and ASD in children, which was supported by previous studies suggesting that parental depression and other psychiatric disorders might be associated with the risk of autism in children[37-39]. When we further looked into those children without paternal affective disorder (the main indication for SSRIs) before birth of child, similar patterns of association to the main analyses were found. Thus, in addition to affective disorders, other indications related to SSRIs use may also contribute to the observed association.

Our study has several methodological strengths. One strength was that the linkage of several nationwide health registries in Denmark enabled us to conduct a large

For peer review only - http://bmjopen.bmj.com/site/about/guidelines.xhtml

cohort study with virtually complete follow-up. The definition on exposure to SSRIs was based on a national registry, which eliminated the risk of recall bias caused by self-report. Another strength was that the information on ASD diagnosis was obtained independently of exposure measurement, which could also mitigate the information bias. Furthermore, the availability of health registry data enabled us to adjust for a number of potential confounders including sociodemographic factors as well as parental psychiatric history.

Limitations need to be considered when interpreting the results of our study. Firstly, we were unable to validate actual use of SSRIs by fathers during the time period of interest because we relied on medical records of dispensed prescriptions. This may lead to misclassification of exposure status because some people may not take the medication or may take it later. Nevertheless, the misclassification was most likely non-differential, which could bias the association toward null. Besides, some patients may receive SSRIs treatment during inpatient admissions which are not included in the prescription registry. We expect this problem to be minor since those inpatients usually have severe psychiatric disorders and are more likely to continue treatment after discharge. Secondly, the 3-month cut-off point was set based on the fact that spermatogenesis takes approximately 70-90 days in humans. Whereas it may be possible that SSRIs drugs induce sperm damage at the very primitive stage. However, the results did not change markedly after extending the putative exposure period to the last 6 months prior to conception (data not shown). Thirdly, ASD in children was ascertained through the DNPR and the DPCRR, which did not include

For peer review only - http://bmjopen.bmj.com/site/about/guidelines.xhtml

those children who received the diagnosis of ASD from private psychiatrists or remained undiagnosed at the end of the follow-up. However, the prevalence of ASD in our cohort was 1.13%, which was similar to that reported in the United States during the study period (1.14%) [40]. Hence the bias introduced by case identification was expected to be minimal.

Conclusions

Our evidence does not support that paternal SSRIs use before conception increases the risk of ASD in the offspring, but implicates that paternal underlying indications related to SSRIs use, or other unmeasured confounding factors may play a role.

Footnotes

This cohort study was approved by the Danish Data Protection Agency (Record No. 2013-41-2569).

Contributors: HL had full access to all the data in the study and took responsibility for the integrity of the data and the accuracy of the data analysis. HL, WY and JL conceptualized and designed the study. FY conducted the statistical analysis. HL, PC and FY interpreted the results and FY drafted the initial manuscript. All authors reviewed the manuscript and approved the final version.

Funding: The study was supported by the National Natural Science Foundation of China (81428011), the National Key Research and Development Program of China (2016YFC1000505), the European Research Council (ERC-2010-StG-260242-PROGEURO), Nordic Cancer Union (2015-176673), Danish

For peer review only - http://bmjopen.bmj.com/site/about/guidelines.xhtml

Council for Independent Research (DFF-6110-00019), and Karen Elise Jensens Fond (2016).

Competing interests: The authors declare that they have no conflict of interest.

Statement of the independence of researchers from funders: The funding sources had no role in the design and conduct of the study; collection, management, analysis, and interpretation of the data; and preparation, review, or approval of the manuscript; and decision to submit the manuscript for publication. The researchers were independent from the funders.

Ethical approval: For this type of study formal consent is not required.

Data sharing statement: No additional data are available.

Reference

1. Organization WH. Depression [fact sheet no. 369]. 2012. As of September. 2015;30.
2. Wen SW, Yang Q, Garner P, et al. Selective serotonin reuptake inhibitors and adverse pregnancy outcomes. American journal of obstetrics and gynecology. 2006;194(4):961-966.
3. Lund N, Pedersen LH, Henriksen TB. Selective serotonin reuptake inhibitor exposure in utero and pregnancy outcomes. Archives of pediatrics & adolescent medicine. 2009;163(10):949-954.
4. Pedersen LH, Henriksen TB, Vestergaard M, et al. Selective serotonin reuptake inhibitors in pregnancy and congenital malformations: population based cohort study. Bmj. 2009;339:b3569.
5. Oberlander TF, Reebye P, Misri S, et al. Externalizing and attentional behaviors in children of depressed mothers treated with a selective serotonin reuptake inhibitor antidepressant during pregnancy. Archives of pediatrics & adolescent medicine. 2007;161(1):22-29.
6. Nulman I, Koren G, Rovet J, et al. Neurodevelopment of children following prenatal exposure to venlafaxine, selective serotonin reuptake inhibitors, or untreated maternal depression. American Journal of Psychiatry. 2012;169(11):1165-1174.
7. Baird G, Simonoff E, Pickles A, et al. Prevalence of disorders of the autism spectrum in a population cohort of children in South Thames: the Special Needs and Autism Project (SNAP). The lancet. 2006;368(9531):210-215.
8. Rutter M. Incidence of autism spectrum disorders: Changes over time and their meaning*. Acta paediatrica. 2005;94(1):2-15.
9. Hill AP, Zuckerman K, Fombonne E. Epidemiology of autism spectrum disorders. Translational Approaches to Autism Spectrum Disorder: Springer 2015:13-38.
10. Newschaffer CJ, Croen LA, Daniels J, et al. The epidemiology of autism spectrum disorders*. Annu Rev Public Health. 2007;28:235-258.

For peer review only - http://bmjopen.bmj.com/site/about/guidelines.xhtml

11. Gidaya NB, Lee BK, Burstyn I, et al. In utero exposure to selective serotonin reuptake inhibitors and risk for autism spectrum disorder. Journal of autism and developmental disorders. 2014;44(10):2558-2567.

12. Croen LA, Grether JK, Yoshida CK, et al. Antidepressant use during pregnancy and childhood autism spectrum disorders. Archives of general psychiatry. 2011;68(11):1104-1112.

13. Rai D, Lee BK, Dalman C, et al. Parental depression, maternal antidepressant use during pregnancy, and risk of autism spectrum disorders: population based case-control study. 2013.

14. Schirm E, Pedersen L, Tobi H, et al. Drug use among fathers around time of conception: two register based surveys from Denmark and The Netherlands. Pharmacoepidemiology and drug safety. 2004;13(9):609-613.

15. Engeland A, Bramness JG, Daltveit AK, et al. Prescription drug use among fathers and mothers before and during pregnancy. A population - based cohort study of 106 000 pregnancies in Norway 2004 - 2006. British journal of clinical pharmacology. 2008;65(5):653-660.

16. Crijns I, Bos J, Knol M, et al. Paternal drug use: before and during pregnancy. Expert opinion on drug safety. 2012;11(4):513-518.

17. Sever LE. Male-mediated developmental toxicity. Toxicology & Applied Pharmacology. 1993;14(1):506–513.

18. Friedler G. Paternal exposures: Impact on reproductive and developmental outcome. An overview. Pharmacology Biochemistry & Behavior. 1996;55(4):691-700.

19. Anderson D, Schmid TE, Baumgartner A. Male-mediated developmental toxicity. Asian journal of andrology. 2014;16(1):81.

20. Cordier S. Evidence for a role of paternal exposures in developmental toxicity. Basic & clinical pharmacology & toxicology. 2008;102(2):176-181.

21. Engeland A, Bjørge T, Daltveit AK, et al. Effects of preconceptional paternal drug exposure on birth outcomes: cohort study of 340 000 pregnancies using Norwegian population - based databases. British journal of clinical pharmacology. 2013;75(4):1134-1141.

22. Tanrikut C, Feldman AS, Altemus M, et al. Adverse effect of paroxetine on sperm. Fertility and sterility. 2010;94(3):1021-1026.

23. Safarinejad MR. Sperm DNA damage and semen quality impairment after treatment with selective serotonin reuptake inhibitors detected using semen analysis and sperm chromatin structure assay. The Journal of urology. 2008;180(5):2124-2128.

24. Lewis S, Aitken R. DNA damage to spermatozoa has impacts on fertilization and pregnancy. Cell and tissue research. 2005;322(1):33-41.

25. Pedersen CB, Gøtzsche H, Møller JØ, et al. The Danish civil registration system. Dan Med Bull. 2006;53(4):441-449.

26. Knudsen LB, Olsen J. The Danish Medical Birth Registry. Danish medical bulletin. 1998;45(3):320-323.

27. Kildemoes HW, Sørensen HT, Hallas J. The Danish national prescription registry. Scandinavian journal of public health. 2011;39(7 suppl):38-41.

28. Mors O, Perto GP, Mortensen PB. The Danish psychiatric central research register. Scandinavian journal of public health. 2011;39(7 suppl):54-57.

29. Andersen TF, Madsen M, Jørgensen J, et al. The Danish National Hospital Register. A valuable source of data for modern health sciences. Danish medical bulletin. 1999;46(3):263-268.

30. Lauritsen MB, Jørgensen M, Madsen KM, et al. Validity of childhood autism in the Danish

For peer review only - http://bmjopen.bmj.com/site/about/guidelines.xhtml

Psychiatric Central Register: findings from a cohort sample born 1990–1999. Journal of autism and developmental disorders. 2010;40(2):139-148.

31. Christensen J, Parner ET, Vestergaard M. Antidepressant exposure in pregnancy and risk of autism spectrum disorders. Clinical epidemiology. 2013;5:449-459.

32. Hviid A, Melbye M, Pasternak B. Use of selective serotonin reuptake inhibitors during pregnancy and risk of autism. New England Journal of Medicine. 2013;369(25):2406-2415.

33. Maciag D, Simpson KL, Coppinger D, et al. Neonatal antidepressant exposure has lasting effects on behavior and serotonin circuitry. Neuropsychopharmacology. 2006;31(1):47-57.

34. Bonnin A, Levitt P. Fetal, maternal and placental sources of serotonin and new implications for developmental programming of the brain. Neuroscience. 2011;197:1.

35. Tanrikut C, Schlegel PN. Antidepressant-associated changes in semen parameters. Urology. 2007;69(1):185. e185-185. e187.

36. Elmazoudy R, Abdelhameed N, Elmasry A. Paternal dapoxetine administration induced deterioration in reproductive performance, fetal outcome, sexual behavior and biochemistry of male rats. International Journal of Impotence Research. 2015;27(6).

37. Daniels JL, Forssen U, Hultman CM, et al. Parental psychiatric disorders associated with autism spectrum disorders in the offspring. Pediatrics. 2008;121(5):e1357-e1362.

38. Larsson HJ, Eaton WW, Madsen KM, et al. Risk factors for autism: perinatal factors, parental psychiatric history, and socioeconomic status. American journal of epidemiology. 2005;161(10):916-925.

39. Jokiranta E, Brown AS, Heinimaa M, et al. Parental psychiatric disorders and autism spectrum disorders. Psychiatry research. 2013;207(3):203-211.

40. Kogan MD, Blumberg SJ, Schieve LA, et al. Prevalence of parent-reported diagnosis of autism spectrum disorder among children in the US, 2007. Pediatrics. 2009;124(5):1395-1403.

For peer review only - http://bmjopen.bmj.com/site/about/guidelines.xhtml

Table1. Baseline Characteristics of the study population

Characteristic	Paternal SSRIs use during the last 3 months prior to conception (N=6,870)	No paternal SSRIs use during the last 3 months prior to conception (N=663, 052)
**Calendar Year of birth, No. (%)**		
1998-2000	1,021(14.9)	185,784(28.0)
2001-2003	1,526(22.2)	179,952(27.1)
2004-2006	2,305(33.5)	178,664(27.0)
2007-2008	2,018(29.4)	118,652(17.9)
**Gender, No. (%)**		
Boy	3,549(51.7)	340,194(51.3)
Girl	3,321(48.3)	322,858(48.7)
**Birth Weight (g), No. (%)**		
<2500	278(4.1)	22,315(3.4)
2500-3250	1,729(25.2)	154,494(23.3)
3250-4000	3,582(52.1)	348,853(52.6)
4000-8000	1,243(18.1)	132,343(19.9)
Unknown	38(0.5)	5,047(0.8)
**Parity, No. (%)**		
1	2,693(39.2)	282,895(42.7)
2	2,433(35.4)	250,496(37.8)
≥3	1,744(25.4)	129,661(19.5)
**Preterm Birth, No. (%)** (<37 weeks)		
No	6,489(94.4)	630,851(95.1)
Yes	381(5.6)	32,201(4.9)
**Apgar score at 5 minutes, No. (%)**		
0-7	91(1.3)	8,224(1.2)
8-9	442(6.4)	39,941(6.0)
10	6,269(91.3)	607,634(91.7)
Unknown	68(1.0)	7,253(1.1)
**Maternal age at child birth (years), No. (%)**		
≤25	958(13.9)	99,273(15.0)
26-30	2,238(32.6)	244,419(36.9)
31-35	2,386(34.7)	225,610(34.0)
>35	1,288(18.8)	93,750(14.1)
**Paternal age at child birth (years), No. (%)**		
≤25	364(5.3)	47,490(7.2)
26-30	1,434(20.9)	180,612(27.2)
31-35	2,395(34.8)	242,702(36.6)
>35	2,677(39.0)	192,248(29.0)

For peer review only

For peer review only - http://bmjopen.bmj.com/site/about/guidelines.xhtml

Table1. Baseline Characteristics of the study population (continued)

**Maternal smoking status[a], No. (%)**		
No	4,326(63.0)	477,574(72.0)
Yes	1,334(19.4)	106,233(16.0)
Unknown	1,210(17.6)	79,245(12.0)
**Maternal AD use during pregnancy, No. (%)**		
No	6,548(94.0)	653,496(98.6)
Yes	412(6.0)	9,556(1.4)
**Maternal history of Affective disorder, No. (%)**		
No	6,607(96.2)	654,234(98.7)
Yes	263(3.8)	8,818(1.3)
**Paternal history of Affective disorder, No. (%)**		
No	6,099 (88.8)	659,639(99.5)
Yes	771(11.2)	3,413(0.5)
**Maternal history of psychiatric disorder, No. (%)**		
No	6,175(89.9)	626,840(94.5)
Yes	695(10.1)	36,212(5.5)
**Paternal history of psychiatric disorder, No. (%)**		
No	5,092 (74.1)	630,9909(95.2)
Yes	1,178(25.9)	32,062(4.8)

Abbreviations: SSRI, selective serotonin reuptake inhibitor; N, number; AD, antidepressant drugs;

[a] Maternal smoking information was missing among those children born from 2007 to 2008.

For peer review only

For peer review only - http://bmjopen.bmj.com/site/about/guidelines.xhtml

Table2. Association between paternal SSRIs use before conception and ASD in offspring

Paternal SSRIs use before conception	Offspring with ASD no.	Follow-up no. of person-yr	Hazard Ratio (95% CI)		
			Crude	Model 1[a]	Model 2[b]
No use during the last 3 months prior to conception	7,473	6,765,205	Ref	Ref	Ref
Use during the last 3 months prior to conception	104	61,555	1.62(1.33-1.96)	1.54(1.27-1.88)	1.43(1.18-1.74)
No use during the last 1 year prior to conception	7,429	6,736,654	Ref	Ref	Ref
Use only from the last 1 year to the last 3 months prior to conception	71	39,422	1.71(1.35-2.16)	1.66(1.31-2.09)	1.54(1.21-1.94)
Use only during the last 3 months prior to conception	20	14,738	1.29(0.83-2.00)	1.24 (0.80-1.93)	1.17(0.75-1.82)
Use both before and during the last 3 months prior to conception	57	35,946	1.52(1.17-1.97)	1.43(1.10-1.86)	1.32(1.02-1.72)

Abbreviations: AD, antidepressant drugs; SSRI, selective serotonin reuptake inhibitor; ASD, Autism Spectrum Disorder; No., number; HR, Hazard Ratio

[a] Adjusted for calendar year of birth, sex, parity, mother age, father age, maternal smoking, mother psychiatric history, maternal AD use during pregnancy

[b] Model 1 further adjusted for father psychiatric history

For peer review only

Table3. Association between paternal SSRIs use before conception and ASD in offspring born to fathers diagnosed with affective disorder or not

Paternal SSRIs use before conception	Offspring with ASD no.	Follow-up no. of person-yr	Hazard Ratio (95% CI)		
			Crude	Model 1[a]	Model 2[b]
**Fathers with affective disorder**					
No use during the last 3 months prior to conception	58	30,908	Ref	Ref	Ref
Use during the last 3 months prior to conception	14	6,625	1.17(0.65-2.09)	1.10(0.61-1.98)	1.10(0.61-1.98)
No use during the last 1 year prior to conception	53	28,033	Ref	Ref	Ref
Use only from the last 1 year to the last 3 months prior to conception	8	4,082	1.07(0.51-2.25)	1.08(0.51-2.30)	1.08(0.51-2.30)
Use only during the last 3 months prior to conception	2	1,164	0.91(0.22-3.75)	0.89(0.21-3.75)	0.89(0.21-3.75)
Use both before and during the last 3 months prior to conception	9	4,254	1.16(0.57-2.35)	1.10(0.55-2.22)	1.10(0.55-2.22)
**Fathers without affective disorder**					
No use during the last 3 months prior to conception	7,415	6,752,899	Ref	Ref	Ref
Use during the last 3 months prior to conception	90	36,329	1.57(1.27-1.93)	1.52(1.23-1.87)	1.45(1.18-1.79)
No use during the last 1 year prior to conception	7,376	6,714,926	Ref	Ref	Ref
Use only from the last 1 year to the last 3 months prior to conception	63	37,973	1.69(1.32-2.17)	1.64(1.28-2.11)	1.57(1.23-2.02)
Use only during the last 3 months prior to conception	18	7,354	1.26(0.79-2.00)	1.23(0.77-1.95)	1.19(0.75-1.88)
Use both before and during the last 3 months prior to conception	48	28,974	1.45(1.09-1.93)	1.38(1.04-1.84)	1.32(0.99-1.76)

Abbreviations: AD, antidepressant drugs; SSRI, selective serotonin reuptake inhibitor; ASD, Autism Spectrum Disorder; No., number; HR, Hazard Ratio

[a] Adjusted for calendar year of birth, parity, mother age, father age, maternal smoking, mother psychiatric history , maternal AD use during pregnancy

[b] Model 1 further adjusted for father psychiatric history

For peer review only - http://bmjopen.bmj.com/site/about/guidelines.xhtml

Table4. Paternal SSRI use before pregnancy and ASD in exposed and unexposed siblings from 5,479 families

Paternal SSRIs use before conception	Number of offspring no.	Offspring with ASD no.	Hazard Ratio (95% CI)	
			Crude	Model 1[a]
No use during the last 3 months prior to conception	2,792	45	Ref	Ref
Use during the last 3 months prior to conception	2,687	23	1.08(1.01-1.16)	1.04(0.94-1.14)

Abbreviations: AD, antidepressant drugs; SSRI, selective serotonin reuptake inhibitor; ASD, Autism Spectrum Disorder; No., number; HR, Hazard Ratio

[a] Adjusted for sex, parity, mother age, father age, maternal smoking, maternal AD use during pregnancy

For peer review only - http://bmjopen.bmj.com/site/about/guidelines.xhtml

TablesS1. Association between paternal SSRIs use before conception and ASD in offspring: analyses stratified by gender

Paternal SSRIs use before conception	Offspring with ASD no.	Follow-up no. of person-yr	Hazard Ratio (95% CI)		
			Crude	Model 1[a]	Model 2[b]
**Boys**					
No use during the last 3 months prior to conception	6,005	3,459,592	Ref	Ref	Ref
Use during the last 3 months prior to conception	84	31,642	1.61(1.30-2.00)	1.55(1.25-1.92)	1.44(1.16-1.79)
No use during the last 1 year prior to conception	5,969	3,444,956	Ref	Ref	Ref
Use only from the last 1 year to the last 3 months prior to conception	58	20,031	1.74(1.34-2.25)	1.69(1.30-2.19)	1.58(1.22-2.05)
Use only during the last 3 months prior to conception	18	7,622	1.42(0.89-2.26)	1.39(0.87-2.21)	1.31(0.83-2.09)
Use both before and during the last 3 months prior to conception	44	18,623	1.44 (1.07-1.93)	1.36(1.01-1.84)	1.26(0.94-1.71)
**Girls**					
No use during the last 3 months prior to conception	1,468	3,305,613	Ref	Ref	Ref
Use during the last 3 months prior to conception	20	29,914	1.63(1.05-2.53)	1.54(0.99-2.40)	1.41(0.90-2.20)
No use during the last 1 year prior to conception	1,460	3,291,698	Ref	Ref	Ref
Use only from the last 1 year to the last 3 months prior to conception	13	19,390	1.61(0.93-2.78)	1.53(0.88-2.64)	1.37(0.79-2.38)
Use only during the last 3 months prior to conception	2	7,116	0.67(0.17-2.69)	0.64(0.16-2.55)	0.59(0.15-2.36)
Use both before and during the last 3 months prior to conception	13	75,323	1.84(1.06-3.17)	1.74(1.01-3.01)	1.59(0.92-2.75)

Abbreviations: AD, antidepressant drugs; SSRI, selective serotonin reuptake inhibitor; ASD, Autism Spectrum Disorder; No., number; HR, Hazard Ratio

[a] Adjusted for calendar year of birth, parity, mother age, father age, maternal smoking, mother psychiatric history, maternal AD use during pregnancy

[b] Model 1 further adjusted for father psychiatric history

For peer review only

TableS2. Association between paternal SSRIs use before conception and ASD in offspring born to mother who neither received antidepressants medication during pregnancy nor had affective disorders before birth of child

Paternal SSRIs use before conception	Offspring with ASD no.	Follow-up no. of person-yr	Hazard Ratio (95% CI)		
			Crude	Model 1[a]	Model 2[b]
No use during the last 3 months prior to conception	7,184	6,627,720	Ref	Ref	Ref
Use during the last 3 months prior to conception	91	57,081	1.55(1.26-1.91)	1.57(1.28-1.93)	1.45(1.18-1.79)
No use during the last 1 year prior to conception	7,144	6,600,852	Ref	Ref	Ref
Use only from the last 1 year to the last 3 months prior to conception	64	37,032	1.67(1.30-2.13)	1.68(1.31-2.15)	1.56(1.22-1.99)
Use only during the last 3 months prior to conception	16	13,634	1.13(0.69-1.85)	1.15(0.71-1.89)	1.08(0.66-1.77)
Use both before and during the last 3 months prior to conception	51	33,283	1.50(1.13-1.97)	1.50(1.13-1.97)	1.38(1.05-1.82)

Abbreviations: AD, antidepressant drugs; SSRI, selective serotonin reuptake inhibitor; ASD, Autism Spectrum Disorder; No., number; HR, Hazard Ratio

[a] Adjusted for calendar year of birth, parity, mother age, father age, maternal smoking, mother psychiatric history

[b] Model 1 further adjusted for father psychiatric history

For peer review only

For peer review only - http://bmjopen.bmj.com/site/about/guidelines.xhtml

STROBE Statement—checklist of items that should be included in reports of observational studies

	Item No	Recommendation	Page No.
**Title and abstract**	1	(*a*) Indicate the study's design with a commonly used term in the title or the abstract	2
		(*b*) Provide in the abstract an informative and balanced summary of what was done and what was found	2-3
**Introduction**			
Background/rationale	2	Explain the scientific background and rationale for the investigation being reported	4
Objectives	3	State specific objectives, including any prespecified hypotheses	5
**Methods**			
Study design	4	Present key elements of study design early in the paper	6
Setting	5	Describe the setting, locations, and relevant dates, including periods of recruitment, exposure, follow-up, and data collection	6-8
Participants	6	(*a*) *Cohort study*—Give the eligibility criteria, and the sources and methods of selection of participants. Describe methods of follow-up<br>*Case-control study*—Give the eligibility criteria, and the sources and methods of case ascertainment and control selection. Give the rationale for the choice of cases and controls<br>*Cross-sectional study*—Give the eligibility criteria, and the sources and methods of selection of participants	6-8
		(*b*) *Cohort study*—For matched studies, give matching criteria and number of exposed and unexposed<br>*Case-control study*—For matched studies, give matching criteria and the number of controls per case	
Variables	7	Clearly define all outcomes, exposures, predictors, potential confounders, and effect modifiers. Give diagnostic criteria, if applicable	6-8
Data sources/ measurement	8*	For each variable of interest, give sources of data and details of methods of assessment (measurement). Describe comparability of assessment methods if there is more than one group	6-8
Bias	9	Describe any efforts to address potential sources of bias	9-10
Study size	10	Explain how the study size was arrived at	10
Quantitative variables	11	Explain how quantitative variables were handled in the analyses. If applicable, describe which groupings were chosen and why	9
Statistical methods	12	(*a*) Describe all statistical methods, including those used to control for confounding	9-10
		(*b*) Describe any methods used to examine subgroups and interactions	9
		(*c*) Explain how missing data were addressed	9
		(*d*) *Cohort study*—If applicable, explain how loss to follow-up was addressed<br>*Case-control study*—If applicable, explain how matching of cases and controls was addressed<br>*Cross-sectional study*—If applicable, describe analytical methods taking account of sampling strategy	Not applicable
		(*e*) Describe any sensitivity analyses	9

For peer review only

**Results**			
Participants	13*	(a) Report numbers of individuals at each stage of study—eg numbers potentially eligible, examined for eligibility, confirmed eligible, included in the study, completing follow-up, and analysed	10
		(b) Give reasons for non-participation at each stage	Not applicable
		(c) Consider use of a flow diagram	
Descriptive data	14*	(a) Give characteristics of study participants (eg demographic, clinical, social) and information on exposures and potential confounders	10
		(b) Indicate number of participants with missing data for each variable of interest	20
		(c) *Cohort study*—Summarise follow-up time (eg, average and total amount)	11
Outcome data	15*	*Cohort study*—Report numbers of outcome events or summary measures over time	10-11
		Case-control study—Report numbers in each exposure category, or summary measures of exposure	
		Cross-sectional study—Report numbers of outcome events or summary measures	
Main results	16	(*a*) Give unadjusted estimates and, if applicable, confounder-adjusted estimates and their precision (eg, 95% confidence interval). Make clear which confounders were adjusted for and why they were included	11
		(*b*) Report category boundaries when continuous variables were categorized	19
		(*c*) If relevant, consider translating estimates of relative risk into absolute risk for a meaningful time period	Not relevant
Other analyses	17	Report other analyses done—eg analyses of subgroups and interactions, and sensitivity analyses	11-12
**Discussion**			
Key results	18	Summarise key results with reference to study objectives	12
Limitations	19	Discuss limitations of the study, taking into account sources of potential bias or imprecision. Discuss both direction and magnitude of any potential bias	14-15
Interpretation	20	Give a cautious overall interpretation of results considering objectives, limitations, multiplicity of analyses, results from similar studies, and other relevant evidence	12-13
Generalisability	21	Discuss the generalisability (external validity) of the study results	14
**Other information**			
Funding	22	Give the source of funding and the role of the funders for the present study and, if applicable, for the original study on which the present article is based	15

*Give information separately for cases and controls in case-control studies and, if applicable, for exposed and unexposed groups in cohort and cross-sectional studies.

Note: An Explanation and Elaboration article discusses each checklist item and gives methodological background and published examples of transparent reporting. The STROBE checklist is best used in conjunction with this article (freely available on the Web sites of PLoS Medicine at http://www.plosmedicine.org/, Annals of Internal Medicine at http://www.annals.org/, and Epidemiology at http://www.epidem.com/). Information on the STROBE Initiative is available at www.strobe-statement.org.

For peer review only

For peer review only - http://bmjopen.bmj.com/site/about/guidelines.xhtml

BMJ Open

Risk of autism spectrum disorder in offspring following paternal use of selective serotonin reuptake inhibitors before conception: a population-based cohort study

Journal:	*BMJ Open*
Manuscript ID	bmjopen-2017-016368.R1
Article Type:	Research
Date Submitted by the Author:	19-May-2017
Complete List of Authors:	Yang, Fen; Shanghai Institute of Planned Parenthood Research, Department of Reproductive Epidemiology and Social Science; Aarhus Universitetshospital, Department of Clinical Epidemiology<br>Chen, Jianping; Shanghai Institute of Planned Parenthood Research, Department of Reproductive Epidemiology and Social Science<br>Miao, Mao-Hua; Shanghai Institute of Planned Parenthood Research, Department of Reproductive Epidemiology and Social Science, National Population and Family Planning Key Laboratory of Contraceptive Drugs and Devices<br>Yuan, Wei; Shanghai Institute of Planned Parenthood Research, Reproductive Epidemiology, National Population and Family Planning Key Laboratory of Contraceptive Drugs and Devices<br>Li, Lin; Shanghai Institute of Planned Parenthood Research<br>Liang, Hong; Shanghai Institute of Planned Parenthood Research, Department of Reproductive Epidemiology and Social Science, National Population and Family Planning Key Laboratory of Contraceptive Drugs and Devices<br>Ehrenstein, Vera; Aarhus Universitetshospital<br>Li, Jiong; Aarhus Universitetshospital, Department of Clinical Epidemiology
**Primary Subject Heading**:	Public health
Secondary Subject Heading:	Mental health
Keywords:	MENTAL HEALTH, Child & adolescent psychiatry < PSYCHIATRY, Adult psychiatry < PSYCHIATRY, NEUROPATHOLOGY

SCHOLARONE™
Manuscripts

Risk of autism spectrum disorder in offspring following paternal use of selective serotonin reuptake inhibitors before conception: a population-based cohort study

Fen Yang[1,2]; Jianping Chen[1]; Maohua Miao[1]; Wei Yuan [1]; Lin Li [1]; Hong Liang[1]; Vera Ehrenstein[2]; Jiong Li [2]

Author Affiliation

[1]Key Lab. of Reproduction Regulation of NPFPC, SIPPR, IRD, Fudan University, Shanghai, 200032, China

[2]Department of Clinical Epidemiology, Aarhus University Hospital, Olof Palmes Alle 43-45, DK-8200 Aarhus N, Denmark

Corresponding Author

Hong Liang, MD, PhD

Key Lab. of Reproduction Regulation of NPFPC, SIPPR, IRD, Fudan University, #779 Lao Hu Min Road, Shanghai, 200032, China.

Email: lucylhcn@163.com;

Phone number: 86 -21-64771683

For peer review only - http://bmjopen.bmj.com/site/about/guidelines.xhtml

Abstract

Objective

The present study aimed to examine the association between paternal selective serotonin reuptake inhibitors (SSRIs) use before conception and the risk of autism spectrum disorder (ASD) in offspring.

Design

A population-based cohort study

Methods

Based on Danish national registers, we conducted a cohort study of 669,922 children born from 1998 to 2008, with follow-up throughout 2013. The children whose fathers used SSRIs during the last 3 months prior to conception were identified as the exposed. Cox regression model was used to estimate the hazard ratio (HR) for ASD in children.

Results

Compared with unexposed children, the exposed had a 1.43-fold higher risk of ASD (HR=1.43, 95% confidence interval [CI]: 1.18-1.74) after adjusting for potential confounders. When extending the exposure window to 1 year before conception, the adjusted HR (aHR) of ASD in children of fathers who used SSRIs only from the last year till the last 3 months prior to conception and who used SSRIs both before and during the last 3 months prior to conception was 1.54 (95%CI: 1.21-1.94) and 1.32 (95%CI: 1.02-1.72), respectively. Whereas, the risk in children of fathers who used SRRIs only during the last 3 months prior to conception decreased and became

For peer review only - http://bmjopen.bmj.com/site/about/guidelines.xhtml

nonsignificant (aHR=1.17, 95%CI: 0.75-1.82).

Conclusions

The increased risk of ASD in the offspring associated with paternal SSRIs use before conception may be attributable to the underlying indications.

Strengths and limitations of this study

- This is the very first study that investigates the link between paternal antidepressants use and ASD risk in children.
- A number of potential confounders, including sociodemographic factors as well as parental psychiatric history, can be adjusted for based on the availability of national health registry data.
- Actual use of SSRIs by fathers during the time period of interest may not be validated.
- Those children who received the diagnosis of ASD from private psychiatrists or remained undiagnosed at the end of the follow-up may not be identified as ASD case.

For peer review only - http://bmjopen.bmj.com/site/about/guidelines.xhtml

Introduction

Depression is a common mental health problem that affects approximately 350 million people worldwide[1]. Selective serotonin reuptake inhibitors (SSRIs) are commonly prescribed for the treatment of depression and other anxiety-related disorders. However, an increasing body of evidence suggests that prenatal exposure to SSRIs is associated with adverse obstetrical and neonatal outcomes[2-5]. In addition, recent studies have indicated a possible link between prenatal SSRIs exposure and neurobehavioral problems in children[2, 6-10], which may be due to the biological mechanisms (i.e. dysfunctional serotonin signaling)[11]. Whereas several studies have suggested that the previously observed association between prenatal SSRIs exposure and neurodevelopmental diseases might due to confounding by related indications[12-15].

Autism spectrum disorder (ASD) is one of the major neurodevelopmental disorders characterized by impairments in reciprocal social interaction, communication, and repetitive or stereotypic behavior[16]. The estimated prevalence of ASD has dramatically increased from less than 1 in 2,000 children in 1960s to approximately 1 in 145 children today[17, 18]. Both genetic and environmental risk factors may contribute to ASD [19]. Recently, maternal use of SSRIs during pregnancy has been reported to be associated with an increased risk of ASD in children[10, 20-23].

If maternal SSRIs exposure during pregnancy plays a role in fetal, even in child's, development, an extended effect of earlier exposure on gamete, particularly human sperm, is biologically plausible. Therefore, the potential role of paternal SSRIs use in

For peer review only - http://bmjopen.bmj.com/site/about/guidelines.xhtml

ASD should also be taken into consideration. Studies in the Nordic countries show that one-third of the fathers used prescription drugs during the last 6 months prior to conception [24] , and approximately 1.4% of fathers were dispensed SSRIs during the last 3 months prior to conception [25, 26]. Although the information regarding the potential effects of SSRI use by fathers is limited, the hypothesis of an increased risk of adverse pregnancy or neonatal outcome associated with paternal drug exposure is not new. Evidence from experimental studies suggests that paternal exposure to a broad range of xenobiotic agents may induce reproductive and developmental abnormalities in the subsequent offspring, e.g., spontaneous abortions, congenital malformations, growth retardation, neurobehavioral deficits, or carcinogenesis[27-29]. Epidemiologic studies have also shown that certain pharmacological agents used by fathers before conception may increase the risk of spontaneous abortions, birth defects or childhood cancers[30, 31]. The potential mechanisms behind the male-mediated effects include: 1) genetic or epigenetic changes with direct disturbances in spermatocytes; 2) indirect effects by transmission of the xenobiotic agents to the female via the seminal fluid[27, 28]. Several studies have indicated the adverse effects of paternal SSRIs use, including impaired semen quality and abnormal sperm DNA fragmentation [32, 33], which has been reported to be associated with diminished fertility, adverse pregnancy outcomes, and an increased risk of childhood disease [34].

Little is known on whether paternal SSRIs use before conception contributes to the risk of ASD in offspring. We conducted a population-based cohort study to examine the association between paternal SSRIs use during the last 3 months prior to

For peer review only - http://bmjopen.bmj.com/site/about/guidelines.xhtml

conception and risk of ASD in offspring, utilizing data from national Danish health registries.

Methods

Study Population

This study was based on several national registers in Denmark. Each Danish resident is assigned a unique personal identification number (a 10-digit civil registration system number used in all registries), which enables accurate linkage of national registries at the individual level[35]. The Danish Medical Birth Registry (DMBR) contains records of all deliveries in Denmark since 1973 and includes information about gestational age at birth from 1978[36].Using the DMBR data, we identified a cohort of all singletons born alive in Denmark during the period of January 1, 1998 through December 31, 2008 (n=687,580). We excluded children without linkage to their father (n=16,955) or their mother (n=74), with missing parity (n=208), and with missing or extreme values of gestational age (≤23 weeks or ≥45 weeks, n=421). A total of 669,922 children were included in the analysis.

Data on SSRIs use

Information on SSRIs use was drawn from the Danish National Prescription Registry (DNPR)[37]. Since 1995, this registry has recorded all redeemed prescriptions in Denmark with the following information: the civil registration number of the patient, the dispensing date, the medication code (the WHO Anatomical Therapeutic Chemical (ATC) classification system), the number of packages prescribed, and the number of

For peer review only - http://bmjopen.bmj.com/site/about/guidelines.xhtml

doses units in package. Selective serotonin reuptake inhibitors use was identified based on ATC codes: fluoxetine (N06AB03), citalopram (N06AB04), paroxetine (N06AB05), sertraline (N06AB06), fluvoxamine (N06AB08), and escitalopram (N06AB10). It is estimated that spermatogenesis takes approximately 74 days[30], therefore we chose the last 3 months prior to conception as the exposure window to cover the susceptible time period. A child was considered exposed if the dispensing date fell within the specified exposure window or the number of days for which the SSRIs medication was supplied overlapped any portion of the exposure window. Children born to fathers who had no prescriptions for SSRIs and no supply overlap during the entire exposure window were considered unexposed. The date of conception was estimated by subtracting gestational age from the date of delivery. Data on paternal SSRIs use during the last two years prior to the conception were extracted for further analyses. We also retrieved the information about maternal SSRIs use during the pregnancy.

Autism spectrum disorders

Autism spectrum disorders in children were identified by using the Danish Psychiatric Central Research Register (DPCRR) and the Danish National Patient Register (DNPR). The DPCRR contains diagnostic information on every admission from psychiatric hospitals and psychiatric wards in general hospitals in Denmark since 1969, and includes data on all outpatient visits and emergency room contacts since 1995[38]. The DNPR has collected data on all inpatients from all somatic hospitals in Denmark since 1977 and outpatients from 1995[39]. The combined data from the two

For peer review only - http://bmjopen.bmj.com/site/about/guidelines.xhtml

registries were used to identify all children diagnosed with ASD. During the study period, the diagnosis of ASD was based on the International Classification of Diseases, 10th version (ICD-10) codes of F84.0 (Childhood autism), F84.1 (Atypical autism), F84.5 (Asperger syndrome), F84.8 (other pervasive developmental disorders) and F84.9 (unspecified pervasive developmental disorders). The quality of the childhood autism diagnosis has been validated and the diagnoses could be verified in 94% of the children with a record in the DPCRR[40]. Children were followed up from birth till first diagnosis of ASD, death, emigration, or December 31, 2013, whichever came first.

Covariates

Using the Danish nationwide health registers, we retrieved data on characteristics that may be associated with offspring ASD or paternal SSRIs use. For each child, we obtained information on calendar year of birth, gender of the child, birth weight, Apgar score at 5 minutes, gestational age, maternal parity, maternal age at child birth, paternal age at child birth and maternal smoking status during pregnancy from the DMBR. Parents' psychiatric history prior to birth of the index child was obtained from DPCRR by ICD-8 codes 290-315 from 1977 to 1993 and ICD-10 codes F00-F99 from 1994 onward. Furthermore, we identified parents diagnosed with affective disorders before birth of child (specifically, ICD-8 codes 296.09, 296.19, 296.29, 296.39, 296.99, 298.09, 298.19, 300.49, and 301.19, and ICD-10 codes F30–F34 and F38–F39) using the DPCRR.

Statistical Analysis

For peer review only - http://bmjopen.bmj.com/site/about/guidelines.xhtml

Cox proportional-hazards regression models were used to estimate the hazard ratios (HRs) and 95% confidence intervals (CIs) of ASD in children following exposure to paternal SSRIs use before conception.

We adjusted for variables as following: the calendar year of birth (1998-2000, 2001-2003, 2004-2006, 2007-2008), gender of the child (boy, girl), parity (1, 2, ≥3), parental age at child birth (≤25, 26-30, 31-35, and >35 years), maternal smoking status during pregnancy (yes or no), maternal history of psychiatric disorders before birth of child (yes or no), maternal antidepressants use during pregnancy (yes or no) in model 1. We additionally adjusted for paternal history of psychiatric disorders before birth of child (yes or no) in model 2. Models were also run with the exclusion of those children with missing data for covariates.

To distinguish the direct effects of SSRIs use from the effects of the indication of SSRIs use, we extended the exposure window to 1 year before conception. We then re-categorized the exposed children into three subgroups: children of fathers who used SSRIs 1) only from the last year till the last 3 months prior to conception (former users); 2) only during the last 3 months prior to conception (current short-term users, hereinafter referred to as 'current users'); 3) both before and during the last 3 months prior to conception (both former and current users). The reference group consisted of those children born to fathers who never used SSRIs medication through the last year before conception.

The stratified analysis was performed to examine whether the association between paternal SSRIs use and ASD in children differed by gender. We also restricted the

For peer review only - http://bmjopen.bmj.com/site/about/guidelines.xhtml

analyses to children whose mothers neither received antidepressant medication during pregnancy nor had affective disorder before birth of child. To further distinguish the effect of SSRIs medication from that of the main indication (i.e., affective disorders) for SSRIs treatment, we performed stratified analyses according to paternal history of affective disorder before birth of the index child. As for those children born to fathers with affective disorders, the ASD risk we examined could be solely attributable to paternal SSRIs use since both of the exposed children and the reference children were with paternal affective disorders.

Besides, to control for unmeasured family-related confounding factors, we re-conducted a sibling study by restricting to families with more than one child and with at least one child with paternal SSRIs preconception exposure. Using the stratified Cox proportional-hazards regression, we then compared exposed siblings to unexposed siblings to estimate the association between paternal SSRIs use before conception and ASD in children.

All analyses were performed using SAS version 9.1 (SAS Institute Inc, Cary, North Carolina, USA).

Results

Among the 669,922 singletons included in the study, 6,870 (1.03%) children were born to fathers who had redeemed a prescription for SSRIs during the last 3 months prior to conception. During the study period, a total of 7,577 children were diagnosed with ASD. The median age at diagnosis of ASD was 6.77 years (interquartile range: 4.84 to 9.48 years). Characteristics of the study population are shown in Table 1.

For peer review only - http://bmjopen.bmj.com/site/about/guidelines.xhtml

Compared with the unexposed group, there were a higher proportion of exposed children born in later calendar years. Fathers in exposed group were more likely to be older at child birth and to have a history of psychiatric disorder (including affective disorder) before birth of child. Mothers of exposed children were characterized as having higher parity, being more likely to be older at child birth, to use antidepressants during pregnancy, and to have a history of psychiatric disorder before birth of child.

Among 61,555 person-years of follow-up, we identified 104 cases of ASD in children born to fathers who used SSRIs during the last 3 months prior to conception (incidence rate: 169 per 100,000 person-years). This incidence rate corresponded to a 62% increased risk of ASD compared with the unexposed group (Table 2). The adjusted HR (aHR) of ASD was 1.54 (95% CI: 1.27-1.88) after adjusting for potential confounders in model 1. After paternal psychiatric history was further adjusted for in model 2, the estimate was 1.43 (95%CI: 1.18-1.74). When extending the exposure window to the last one year prior to conception, after the full adjustment, the HR for ASD in children of fathers who were former users only and who were both former and current users was 1.54 (95%CI: 1.21-1.94) and 1.32 (95%CI: 1.02-1.72), respectively. However, the increased risk decreased and became nonsignificant among children of fathers who were only current users (aHR=1.17, 95%CI: 0.75-1.82, model 2, table2).

The risk estimates of ASD were similar for both boys and girls, regardless of the exposure (Table S1). When we restricted analyses to children whose mothers neither

For peer review only - http://bmjopen.bmj.com/site/about/guidelines.xhtml

used SSRIs during pregnancy nor had affective disorder before birth of child, the results did not change essentially (Table S2).

When the analyses were stratified by paternal affective disorder before birth of the index child, in children born to fathers with a history of affective disorder, there was no association between paternal SSRIs use before conception and ASD in the offspring (model 2: aHR=1.10, 95%CI: 0.61-1.98). For children whose father without affective disorder, the patterns of associations remained similar to those of the main analyses (Table 3).

In the sibling analysis, we identified 5,479 families with more than one child and with at least one child with paternal SSRIs use before conception (Table 4). The risk of ASD in exposed children was similar when compared with their unexposed siblings (aHR=1.04, 95%CI: 0.94-1.14).

Discussion

In this large population-based cohort study, we observed an increased risk of ASD in the offspring following paternal use of SSRIs during the last 3 months prior to conception. However, the risk attenuated after adjusting for a number of potential confounders, especially fathers' psychiatric conditions. When extending the exposure window to one year before conception, the ASD risk persisted among children born to former users but not current users. In addition, among children born to father with affective disorder, no association was observed. Finally, we performed a sibling analysis which allowed for better control of unmeasured familial confounding and no difference in the ASD risk was found among exposed children and their unexposed

For peer review only - http://bmjopen.bmj.com/site/about/guidelines.xhtml

siblings. Taken as a whole, our results did not support that paternal SSRIs use before conception could increase the risk of ASD in children.

Recently, concerns have been raised regarding the risk of ASD in the offspring associated with prenatal exposure to SSRIs. Three previous human studies have suggested that in utero exposure to antidepressants would increase the risk of ASD in children[21-23], although another two studies using the Danish registers have reported no significant association [41, 42]. Basic neuro-biologic studies have showed that prenatal SSRIs administration may be part of a causal pathway to ASD by operating directly on the developing brain[43, 44]. Considering the detrimental effect of SSRIs on sperm [32, 33, 45], paternal SSRIs use before conception may also cause adverse effects on pregnancy outcomes. However, till now, only one study have suggested that SSRIs administration to male rats could induce deterioration in fertility and fetal outcomes (including weight gain, organ weights and feed consumption)[46]. To our knowledge, our study is the first to investigate the link between paternal antidepressants use and ASD risk in children. Considering the limited number of ASD cases and the challenges posed by confounding, further studies are needed to corroborate the findings.

In the present study, the increased risk of ASD observed in former users but not in current users implied that indications for paternal SSRIs use may account for the observed association between paternal SSRIs use and ASD in children, which was supported by previous studies suggesting that parental depression and other psychiatric disorders might be associated with the risk of autism in children[47-49]. In

For peer review only - http://bmjopen.bmj.com/site/about/guidelines.xhtml

children born to fathers without affective disorders, there was an increased risk associated with paternal SSRIs use. Most fathers might receive SSRIs treatment from their general practitioner and were therefore not registered with a diagnosis of affective disorders in the hospital system. Therefore, it was possible that the increased risk associated with prenatal SSRIs use was partly confounded by paternal affective disorders diagnosed outside a hospital department for which we were not able to adjust. In addition, other psychiatric diseases related to SSRIs use might also contribute to the observed association.

Our study has several methodological strengths. One strength was that the linkage of several nationwide health registries in Denmark enabled us to conduct a large cohort study with virtually complete follow-up. The definition on exposure to SSRIs was based on a national registry, which eliminated the risk of recall bias caused by self-report. Another strength was that the information on ASD diagnosis was obtained independently of exposure measurement, which could also mitigate the information bias. Furthermore, the availability of health registry data enabled us to adjust for a number of potential confounders including sociodemographic factors as well as parental psychiatric history.

Limitations need to be considered when interpreting the results of our study. Firstly, we were unable to validate actual use of SSRIs by fathers during the time period of interest because we relied on medical records of dispensed prescriptions. This may lead to misclassification of exposure status because some people may not take the medication or may take it later. Nevertheless, the misclassification was most

For peer review only - http://bmjopen.bmj.com/site/about/guidelines.xhtml

likely non-differential, which could bias the association toward null. Besides, some patients may receive SSRIs treatment during inpatient admissions which are not included in the prescription registry. We expect this problem to be minor since those inpatients usually have severe psychiatric disorders and are more likely to continue treatment after discharge. Secondly, the 3-month cut-off point was set based on the fact that spermatogenesis takes approximately 70-90 days in humans. Whereas it may be possible that SSRIs drugs induce sperm damage at the very primitive stage. However, the results did not change markedly after extending the putative exposure period to the last 6 months prior to conception (data not shown). Thirdly, ASD in children was ascertained through the DNPR and the DPCRR, which did not include those children who received the diagnosis of ASD from private psychiatrists or remained undiagnosed at the end of the follow-up. However, the prevalence of ASD in our cohort was 1.13%, which was similar to that reported in the United States during the study period (1.14%) [50]. Hence the bias introduced by case identification was expected to be minimal.

Conclusions

In the study, paternal SSRI use before conception was associated with an increased risk of ASD in the offspring, especially in the former users who took SSRIs over the longer term. However, null association were observed in exposed children with paternal affective disorders, and similar ASD risk were observed among exposed and unexposed siblings, which implicates that paternal underlying indications related to SSRIs use or other unmeasured confounding factors may explain the increased risk.

For peer review only - http://bmjopen.bmj.com/site/about/guidelines.xhtml

For peer review only

Footnotes

This cohort study was approved by the Danish Data Protection Agency (Document No. 2013-41-2569).

Contributors: HL had full access to all the data in the study and took responsibility for the integrity of the data and the accuracy of the data analysis. HL, WY and JL conceptualized and designed the study. FY conducted the statistical analysis. HL, PC and FY interpreted the results and FY drafted the initial manuscript. All authors reviewed the manuscript and approved the final version.

Funding: The study was supported by the National Natural Science Foundation of China (81428011), the National Key Research and Development Program of China (2016YFC1000505), the European Research Council (ERC-2010-StG-260242-PROGEURO), Nordic Cancer Union (176673,186200), Danish Council for Independent Research (DFF-6110-00019), Karen Elise Jensens Fond (2016), and the Program for Clinical Research Infrastructure (PROCRIN) established by the Lundbeck Foundation and the Novo Nordisk Foundation.

Competing interests: The authors declare that they have no conflict of interest.

Statement of the independence of researchers from funders: The funding sources had no role in the design and conduct of the study; collection, management, analysis, and interpretation of the data; and preparation, review, or approval of the manuscript; and decision to submit the manuscript for publication. The researchers were independent from the funders.

Ethical approval: The study was based on secondary data. No individuals were approached as a result of the study, nor did we access any other data from the

For peer review only

For peer review only - http://bmjopen.bmj.com/site/about/guidelines.xhtml

participants. This study was approved by the Danish Data Protection Agency (Document No. 2013-41-2569). All procedures performed in the study involving human participants were in accordance with the ethical standards of the institutional and/or national research committee and with the 1964 Helsinki declaration and its later amendments or comparable ethical standards. For this type of study formal consent is not required.

Data sharing statement: No additional data are available.

Reference

1. Organization WH. Depression [fact sheet no. 369]. 2012. As of September. 2015;30.
2. Alwan S, Friedman JM, Chambers C. Safety of selective serotonin reuptake inhibitors in pregnancy: a review of current evidence. CNS drugs. 2016;30(6):499-515.
3. Wen SW, Yang Q, Garner P, et al. Selective serotonin reuptake inhibitors and adverse pregnancy outcomes. American journal of obstetrics and gynecology. 2006;194(4):961-966.
4. Lund N, Pedersen LH, Henriksen TB. Selective serotonin reuptake inhibitor exposure in utero and pregnancy outcomes. Archives of pediatrics & adolescent medicine. 2009;163(10):949-954.
5. Pedersen LH, Henriksen TB, Vestergaard M, et al. Selective serotonin reuptake inhibitors in pregnancy and congenital malformations: population based cohort study. Bmj. 2009;339:b3569.
6. Oberlander TF, Reebye P, Misri S, et al. Externalizing and attentional behaviors in children of depressed mothers treated with a selective serotonin reuptake inhibitor antidepressant during pregnancy. Archives of pediatrics & adolescent medicine. 2007;161(1):22-29.
7. Nulman I, Koren G, Rovet J, et al. Neurodevelopment of children following prenatal exposure to venlafaxine, selective serotonin reuptake inhibitors, or untreated maternal depression. American Journal of Psychiatry. 2012;169(11):1165-1174.
8. Healy D, Le Noury J, Mangin D. Links between serotonin reuptake inhibition during pregnancy and neurodevelopmental delay/spectrum disorders: A systematic review of epidemiological and physiological evidence. International Journal of Risk & Safety in Medicine. 2016;28(3):125-141.
9. Clements CC, Castro VM, Blumenthal SR, et al. Prenatal antidepressant exposure is associated with risk for attention-deficit hyperactivity disorder but not autism spectrum disorder in a large health system. Molecular psychiatry. 2015;20(6):727-734.
10. Boukhris T, Sheehy O, Mottron L, et al. Antidepressant use during pregnancy and the risk of autism spectrum disorder in children. JAMA pediatrics. 2016;170(2):117-124.
11. Hadjikhani N. Serotonin, pregnancy and increased autism prevalence: is there a link? Medical Hypotheses. 2010;74(5):880-883.
12. Brown HK, Ray JG, Wilton AS, et al. Association Between Serotonergic Antidepressant Use During Pregnancy and Autism Spectrum Disorder in Children. Jama. 2017;317(15):1544-1552.
13. Sujan AC, Rickert ME, Öberg AS, et al. Associations of Maternal Antidepressant Use During the

For peer review only - http://bmjopen.bmj.com/site/about/guidelines.xhtml

First Trimester of Pregnancy With Preterm Birth, Small for Gestational Age, Autism Spectrum Disorder, and Attention-Deficit/Hyperactivity Disorder in Offspring. Jama. 2017;317(15):1553-1562.
14. Oberlander TF, Zwaigenbaum L. Disentangling Maternal Depression and Antidepressant Use During Pregnancy as Risks for Autism in Children. Jama. 2017;317(15):1533-1534.
15. Mezzacappa A, Lasica P-A, Gianfagna F, et al. Risk for Autism Spectrum Disorders According to Period of Prenatal Antidepressant Exposure: A Systematic Review and Meta-analysis. JAMA pediatrics. 2017.
16. Baird G, Simonoff E, Pickles A, et al. Prevalence of disorders of the autism spectrum in a population cohort of children in South Thames: the Special Needs and Autism Project (SNAP). The lancet. 2006;368(9531):210-215.
17. Rutter M. Incidence of autism spectrum disorders: Changes over time and their meaning*. Acta paediatrica. 2005;94(1):2-15.
18. Hill AP, Zuckerman K, Fombonne E. Epidemiology of autism spectrum disorders. Translational Approaches to Autism Spectrum Disorder: Springer 2015:13-38.
19. Newschaffer CJ, Croen LA, Daniels J, et al. The epidemiology of autism spectrum disorders*. Annu Rev Public Health. 2007;28:235-258.
20. Brown HK, Hussain-Shamsy N, Lunsky Y, et al. The association between antenatal exposure to selective serotonin reuptake inhibitors and autism: a systematic review and meta-analysis. The Journal of clinical psychiatry. 2017;78(1):e48.
21. Gidaya NB, Lee BK, Burstyn I, et al. In utero exposure to selective serotonin reuptake inhibitors and risk for autism spectrum disorder. Journal of autism and developmental disorders. 2014;44(10):2558-2567.
22. Croen LA, Grether JK, Yoshida CK, et al. Antidepressant use during pregnancy and childhood autism spectrum disorders. Archives of general psychiatry. 2011;68(11):1104-1112.
23. Rai D, Lee BK, Dalman C, et al. Parental depression, maternal antidepressant use during pregnancy, and risk of autism spectrum disorders: population based case-control study. 2013.
24. Schirm E, Pedersen L, Tobi H, et al. Drug use among fathers around time of conception: two register based surveys from Denmark and The Netherlands. Pharmacoepidemiology and drug safety. 2004;13(9):609-613.
25. Engeland A, Bramness JG, Daltveit AK, et al. Prescription drug use among fathers and mothers before and during pregnancy. A population‐based cohort study of 106 000 pregnancies in Norway 2004–2006. British journal of clinical pharmacology. 2008;65(5):653-660.
26. Crijns I, Bos J, Knol M, et al. Paternal drug use: before and during pregnancy. Expert opinion on drug safety. 2012;11(4):513-518.
27. Sever LE. Male-mediated developmental toxicity. Toxicology & Applied Pharmacology. 1993;14(1):506–513.
28. Friedler G. Paternal exposures: Impact on reproductive and developmental outcome. An overview. Pharmacology Biochemistry & Behavior. 1996;55(4):691-700.
29. Anderson D, Schmid TE, Baumgartner A. Male-mediated developmental toxicity. Asian journal of andrology. 2014;16(1):81.
30. Cordier S. Evidence for a role of paternal exposures in developmental toxicity. Basic & clinical pharmacology & toxicology. 2008;102(2):176-181.
31. Engeland A, Bjørge T, Daltveit AK, et al. Effects of preconceptional paternal drug exposure on birth outcomes: cohort study of 340 000 pregnancies using Norwegian population‐based databases.

For peer review only
For peer review only - http://bmjopen.bmj.com/site/about/guidelines.xhtml

British journal of clinical pharmacology. 2013;75(4):1134-1141.
32. Tanrikut C, Feldman AS, Altemus M, et al. Adverse effect of paroxetine on sperm. Fertility and sterility. 2010;94(3):1021-1026.
33. Safarinejad MR. Sperm DNA damage and semen quality impairment after treatment with selective serotonin reuptake inhibitors detected using semen analysis and sperm chromatin structure assay. The Journal of urology. 2008;180(5):2124-2128.
34. Lewis S, Aitken R. DNA damage to spermatozoa has impacts on fertilization and pregnancy. Cell and tissue research. 2005;322(1):33-41.
35. Pedersen CB, Gøtzsche H, Møller JØ, et al. The Danish civil registration system. Dan Med Bull. 2006;53(4):441-449.
36. Knudsen LB, Olsen J. The Danish Medical Birth Registry. Danish medical bulletin. 1998;45(3):320-323.
37. Kildemoes HW, Sørensen HT, Hallas J. The Danish national prescription registry. Scandinavian journal of public health. 2011;39(7 suppl):38-41.
38. Mors O, Perto GP, Mortensen PB. The Danish psychiatric central research register. Scandinavian journal of public health. 2011;39(7 suppl):54-57.
39. Andersen TF, Madsen M, Jørgensen J, et al. The Danish National Hospital Register. A valuable source of data for modern health sciences. Danish medical bulletin. 1999;46(3):263-268.
40. Lauritsen MB, Jørgensen M, Madsen KM, et al. Validity of childhood autism in the Danish Psychiatric Central Register: findings from a cohort sample born 1990–1999. Journal of autism and developmental disorders. 2010;40(2):139-148.
41. Christensen J, Parner ET, Vestergaard M. Antidepressant exposure in pregnancy and risk of autism spectrum disorders. Clinical epidemiology. 2013;5:449-459.
42. Hviid A, Melbye M, Pasternak B. Use of selective serotonin reuptake inhibitors during pregnancy and risk of autism. New England Journal of Medicine. 2013;369(25):2406-2415.
43. Maciag D, Simpson KL, Coppinger D, et al. Neonatal antidepressant exposure has lasting effects on behavior and serotonin circuitry. Neuropsychopharmacology. 2006;31(1):47-57.
44. Bonnin A, Levitt P. Fetal, maternal and placental sources of serotonin and new implications for developmental programming of the brain. Neuroscience. 2011;197:1.
45. Tanrikut C, Schlegel PN. Antidepressant-associated changes in semen parameters. Urology. 2007;69(1):185. e185-185. e187.
46. Elmazoudy R, Abdelhameed N, Elmasry A. Paternal dapoxetine administration induced deterioration in reproductive performance, fetal outcome, sexual behavior and biochemistry of male rats. International Journal of Impotence Research. 2015;27(6).
47. Daniels JL, Forssen U, Hultman CM, et al. Parental psychiatric disorders associated with autism spectrum disorders in the offspring. Pediatrics. 2008;121(5):e1357-e1362.
48. Larsson HJ, Eaton WW, Madsen KM, et al. Risk factors for autism: perinatal factors, parental psychiatric history, and socioeconomic status. American journal of epidemiology. 2005;161(10):916-925.
49. Jokiranta E, Brown AS, Heinimaa M, et al. Parental psychiatric disorders and autism spectrum disorders. Psychiatry research. 2013;207(3):203-211.
50. Kogan MD, Blumberg SJ, Schieve LA, et al. Prevalence of parent-reported diagnosis of autism spectrum disorder among children in the US, 2007. Pediatrics. 2009;124(5):1395-1403.

For peer review only - http://bmjopen.bmj.com/site/about/guidelines.xhtml

For peer review only

For peer review only - http://bmjopen.bmj.com/site/about/guidelines.xhtml

Table1. Baseline Characteristics of the study population

Characteristic	Paternal SSRIs use during the last 3 months prior to conception (N=6,870)	No paternal SSRIs use during the last 3 months prior to conception (N=663, 052)
**Calendar Year of birth, No. (%)**		
1998-2000	1,021(14.9)	185,784(28.0)
2001-2003	1,526(22.2)	179,952(27.1)
2004-2006	2,305(33.5)	178,664(27.0)
2007-2008	2,018(29.4)	118,652(17.9)
**Gender, No. (%)**		
Boy	3,549(51.7)	340,194(51.3)
Girl	3,321(48.3)	322,858(48.7)
**Birth Weight (g), No. (%)**		
<2500	278(4.1)	22,315(3.4)
2500-3250	1,729(25.2)	154,494(23.3)
3250-4000	3,582(52.1)	348,853(52.6)
4000-8000	1,243(18.1)	132,343(19.9)
Unknown	38(0.5)	5,047(0.8)
**Parity, No. (%)**		
1	2,693(39.2)	282,895(42.7)
2	2,433(35.4)	250,496(37.8)
≥3	1,744(25.4)	129,661(19.5)
**Preterm Birth, No. (%)** (<37 weeks)		
No	6,489(94.4)	630,851(95.1)
Yes	381(5.6)	32,201(4.9)
**Apgar score at 5 minutes, No. (%)**		
0-7	91(1.3)	8,224(1.2)
8-9	442(6.4)	39,941(6.0)
10	6,269(91.3)	607,634(91.7)
Unknown	68(1.0)	7,253(1.1)
**Maternal age at child birth (years), No. (%)**		
≤25	958(13.9)	99,273(15.0)
26-30	2,238(32.6)	244,419(36.9)
31-35	2,386(34.7)	225,610(34.0)
>35	1,288(18.8)	93,750(14.1)
**Paternal age at child birth (years), No. (%)**		
≤25	364(5.3)	47,490(7.2)
26-30	1,434(20.9)	180,612(27.2)
31-35	2,395(34.8)	242,702(36.6)
>35	2,677(39.0)	192,248(29.0)

For peer review only

For peer review only - http://bmjopen.bmj.com/site/about/guidelines.xhtml

Table1. Baseline Characteristics of the study population (continued)

**Maternal smoking status[a], No. (%)**		
No	4,326(63.0)	477,574(72.0)
Yes	1,334(19.4)	106,233(16.0)
Unknown	1,210(17.6)	79,245(12.0)
**Maternal AD use during pregnancy, No. (%)**		
No	6,548(94.0)	653,496(98.6)
Yes	412(6.0)	9,556(1.4)
**Maternal history of Affective disorder, No. (%)**		
No	6,607(96.2)	654,234(98.7)
Yes	263(3.8)	8,818(1.3)
**Paternal history of Affective disorder, No. (%)**		
No	6,099 (88.8)	659,639(99.5)
Yes	771(11.2)	3,413(0.5)
**Maternal history of psychiatric disorder, No. (%)**		
No	6,175(89.9)	626,840(94.5)
Yes	695(10.1)	36,212(5.5)
**Paternal history of psychiatric disorder, No. (%)**		
No	5,092 (74.1)	630,9909(95.2)
Yes	1,178(25.9)	32,062(4.8)

Abbreviations: SSRI, selective serotonin reuptake inhibitor; N, number; AD, antidepressant drugs;

[a] Maternal smoking information was missing among those children born from 2007 to 2008.

For peer review only

For peer review only - http://bmjopen.bmj.com/site/about/guidelines.xhtml

Table2. Association between paternal SSRIs use before conception and ASD in offspring

Paternal SSRIs use before conception	Offspring with ASD no.	Follow-up no. of person-yr	Hazard Ratio (95% CI)		
			Crude	Model 1[a]	Model 2[b]
No use during the last 3 months prior to conception	7,473	6,765,205	Ref	Ref	Ref
Use during the last 3 months prior to conception	104	61,555	1.62(1.33-1.96)	1.54(1.27-1.88)	1.43(1.18-1.74)
No use during the last 1 year prior to conception	7,429	6,736,654	Ref	Ref	Ref
Use only from the last 1 year to the last 3 months prior to conception	71	39,422	1.71(1.35-2.16)	1.66(1.31-2.09)	1.54(1.21-1.94)
Use only during the last 3 months prior to conception	20	14,738	1.29(0.83-2.00)	1.24 (0.80-1.93)	1.17(0.75-1.82)
Use both before and during the last 3 months prior to conception	57	35,946	1.52(1.17-1.97)	1.43(1.10-1.86)	1.32(1.02-1.72)

Abbreviations: AD, antidepressant drugs; SSRI, selective serotonin reuptake inhibitor; ASD, Autism Spectrum Disorder; No., number; HR, Hazard Ratio

[a] Adjusted for calendar year of birth, sex, parity, mother age, father age, maternal smoking, mother psychiatric history, maternal AD use during pregnancy

[b] Model 1 further adjusted for father psychiatric history

For peer review only

Table3. Association between paternal SSRIs use before conception and ASD in offspring born to fathers diagnosed with affective disorder or not

Paternal SSRIs use before conception	Offspring with ASD no.	Follow-up no. of person-yr	Hazard Ratio (95% CI)		
			Crude	Model 1[a]	Model 2[b]
**Fathers with affective disorder**					
No use during the last 3 months prior to conception	58	30,908	Ref	Ref	Ref
Use during the last 3 months prior to conception	14	6,625	1.17(0.65-2.09)	1.10(0.61-1.98)	1.10(0.61-1.98)
No use during the last 1 year prior to conception	53	28,033	Ref	Ref	Ref
Use only from the last 1 year to the last 3 months prior to conception	8	4,082	1.07(0.51-2.25)	1.08(0.51-2.30)	1.08(0.51-2.30)
Use only during the last 3 months prior to conception	2	1,164	0.91(0.22-3.75)	0.89(0.21-3.75)	0.89(0.21-3.75)
Use both before and during the last 3 months prior to conception	9	4,254	1.16(0.57-2.35)	1.10(0.55-2.22)	1.10(0.55-2.22)
**Fathers without affective disorder**					
No use during the last 3 months prior to conception	7,415	6,752,899	Ref	Ref	Ref
Use during the last 3 months prior to conception	90	36,329	1.57(1.27-1.93)	1.52(1.23-1.87)	1.45(1.18-1.79)
No use during the last 1 year prior to conception	7,376	6,714,926	Ref	Ref	Ref
Use only from the last 1 year to the last 3 months prior to conception	63	37,973	1.69(1.32-2.17)	1.64(1.28-2.11)	1.57(1.23-2.02)
Use only during the last 3 months prior to conception	18	7,354	1.26(0.79-2.00)	1.23(0.77-1.95)	1.19(0.75-1.88)
Use both before and during the last 3 months prior to conception	48	28,974	1.45(1.09-1.93)	1.38(1.04-1.84)	1.32(0.99-1.76)

Abbreviations: AD, antidepressant drugs; SSRI, selective serotonin reuptake inhibitor; ASD, Autism Spectrum Disorder; No., number; HR, Hazard Ratio

[a] Adjusted for calendar year of birth, parity, mother age, father age, maternal smoking, mother psychiatric history , maternal AD use during pregnancy

[b] Model 1 further adjusted for father psychiatric history

For peer review only

For peer review only - http://bmjopen.bmj.com/site/about/guidelines.xhtml

Table4. Paternal SSRI use before pregnancy and ASD in exposed and unexposed siblings from 5,479 families

Paternal SSRIs use before conception	Number of offspring no.	Offspring with ASD no.	Hazard Ratio (95% CI)	
			Crude	Model 1[a]
No use during the last 3 months prior to conception	2,792	45	Ref	Ref
Use during the last 3 months prior to conception	2,687	23	1.08(1.01-1.16)	1.04(0.94-1.14)

Abbreviations: AD, antidepressant drugs; SSRI, selective serotonin reuptake inhibitor; ASD, Autism Spectrum Disorder; No., number; HR, Hazard Ratio

[a] Adjusted for sex, parity, mother age, father age, maternal smoking, maternal AD use during pregnancy

For peer review only

For peer review only - http://bmjopen.bmj.com/site/about/guidelines.xhtml

TablesS1. Association between paternal SSRIs use before conception and ASD in offspring: analyses stratified by gender

Paternal SSRIs use before conception	Offspring with ASD no.	Follow-up no. of person-yr	Hazard Ratio (95% CI)		
			Crude	Model 1[a]	Model 2[b]
**Boys**					
No use during the last 3 months prior to conception	6,005	3,459,592	Ref	Ref	Ref
Use during the last 3 months prior to conception	84	31,642	1.61(1.30-2.00)	1.55(1.25-1.92)	1.44(1.16-1.79)
No use during the last 1 year prior to conception	5,969	3,444,956	Ref	Ref	Ref
Use only from the last 1 year to the last 3 months prior to conception	58	20,031	1.74(1.34-2.25)	1.69(1.30-2.19)	1.58(1.22-2.05)
Use only during the last 3 months prior to conception	18	7,622	1.42(0.89-2.26)	1.39(0.87-2.21)	1.31(0.83-2.09)
Use both before and during the last 3 months prior to conception	44	18,623	1.44 (1.07-1.93)	1.36(1.01-1.84)	1.26(0.94-1.71)
**Girls**					
No use during the last 3 months prior to conception	1,468	3,305,613	Ref	Ref	Ref
Use during the last 3 months prior to conception	20	29,914	1.63(1.05-2.53)	1.54(0.99-2.40)	1.41(0.90-2.20)
No use during the last 1 year prior to conception	1,460	3,291,698	Ref	Ref	Ref
Use only from the last 1 year to the last 3 months prior to conception	13	19,390	1.61(0.93-2.78)	1.53(0.88-2.64)	1.37(0.79-2.38)
Use only during the last 3 months prior to conception	2	7,116	0.67(0.17-2.69)	0.64(0.16-2.55)	0.59(0.15-2.36)
Use both before and during the last 3 months prior to conception	13	75,323	1.84(1.06-3.17)	1.74(1.01-3.01)	1.59(0.92-2.75)

Abbreviations: AD, antidepressant drugs; SSRI, selective serotonin reuptake inhibitor; ASD, Autism Spectrum Disorder; No., number; HR, Hazard Ratio

[a] Adjusted for calendar year of birth, parity, mother age, father age, maternal smoking, mother psychiatric history, maternal AD use during pregnancy

[b] Model 1 further adjusted for father psychiatric history

For peer review only - http://bmjopen.bmj.com/site/about/guidelines.xhtml

For peer review only

TableS2. Association between paternal SSRIs use before conception and ASD in offspring born to mother who neither received antidepressants medication during pregnancy nor had affective disorders before birth of child

Paternal SSRIs use before conception	Offspring with ASD no.	Follow-up no. of person-yr	Hazard Ratio (95% CI)		
			Crude	Model 1[a]	Model 2[b]
No use during the last 3 months prior to conception	7,184	6,627,720	Ref	Ref	Ref
Use during the last 3 months prior to conception	91	57,081	1.55(1.26-1.91)	1.57(1.28-1.93)	1.45(1.18-1.79)
No use during the last 1 year prior to conception	7,144	6,600,852	Ref	Ref	Ref
Use only from the last 1 year to the last 3 months prior to conception	64	37,032	1.67(1.30-2.13)	1.68(1.31-2.15)	1.56(1.22-1.99)
Use only during the last 3 months prior to conception	16	13,634	1.13(0.69-1.85)	1.15(0.71-1.89)	1.08(0.66-1.77)
Use both before and during the last 3 months prior to conception	51	33,283	1.50(1.13-1.97)	1.50(1.13-1.97)	1.38(1.05-1.82)

Abbreviations: AD, antidepressant drugs; SSRI, selective serotonin reuptake inhibitor; ASD, Autism Spectrum Disorder; No., number; HR, Hazard Ratio

[a] Adjusted for calendar year of birth, parity, mother age, father age, maternal smoking, mother psychiatric history

[b] Model 1 further adjusted for father psychiatric history

For peer review only

STROBE Statement—checklist of items that should be included in reports of observational studies

	Item No	Recommendation	Page No.
**Title and abstract**	1	(*a*) Indicate the study's design with a commonly used term in the title or the abstract	2
		(*b*) Provide in the abstract an informative and balanced summary of what was done and what was found	2-3
**Introduction**			
Background/rationale	2	Explain the scientific background and rationale for the investigation being reported	4
Objectives	3	State specific objectives, including any prespecified hypotheses	5
**Methods**			
Study design	4	Present key elements of study design early in the paper	6
Setting	5	Describe the setting, locations, and relevant dates, including periods of recruitment, exposure, follow-up, and data collection	6-8
Participants	6	(*a*) *Cohort study*—Give the eligibility criteria, and the sources and methods of selection of participants. Describe methods of follow-up<br>*Case-control study*—Give the eligibility criteria, and the sources and methods of case ascertainment and control selection. Give the rationale for the choice of cases and controls<br>*Cross-sectional study*—Give the eligibility criteria, and the sources and methods of selection of participants	6-8
		(*b*) *Cohort study*—For matched studies, give matching criteria and number of exposed and unexposed<br>*Case-control study*—For matched studies, give matching criteria and the number of controls per case	
Variables	7	Clearly define all outcomes, exposures, predictors, potential confounders, and effect modifiers. Give diagnostic criteria, if applicable	6-8
Data sources/ measurement	8*	For each variable of interest, give sources of data and details of methods of assessment (measurement). Describe comparability of assessment methods if there is more than one group	6-8
Bias	9	Describe any efforts to address potential sources of bias	9-10
Study size	10	Explain how the study size was arrived at	10
Quantitative variables	11	Explain how quantitative variables were handled in the analyses. If applicable, describe which groupings were chosen and why	9
Statistical methods	12	(*a*) Describe all statistical methods, including those used to control for confounding	9-10
		(*b*) Describe any methods used to examine subgroups and interactions	9
		(*c*) Explain how missing data were addressed	9
		(*d*) *Cohort study*—If applicable, explain how loss to follow-up was addressed<br>*Case-control study*—If applicable, explain how matching of cases and controls was addressed<br>*Cross-sectional study*—If applicable, describe analytical methods taking account of sampling strategy	Not applicable
		(*e*) Describe any sensitivity analyses	9

For peer review only - http://bmjopen.bmj.com/site/about/guidelines.xhtml

**Results**			
Participants	13*	(a) Report numbers of individuals at each stage of study—eg numbers potentially eligible, examined for eligibility, confirmed eligible, included in the study, completing follow-up, and analysed	10
		(b) Give reasons for non-participation at each stage	Not applicable
		(c) Consider use of a flow diagram	
Descriptive data	14*	(a) Give characteristics of study participants (eg demographic, clinical, social) and information on exposures and potential confounders	10
		(b) Indicate number of participants with missing data for each variable of interest	20
		(c) *Cohort study*—Summarise follow-up time (eg, average and total amount)	11
Outcome data	15*	*Cohort study*—Report numbers of outcome events or summary measures over time	10-11
		Case-control study—Report numbers in each exposure category, or summary measures of exposure	
		Cross-sectional study—Report numbers of outcome events or summary measures	
Main results	16	(*a*) Give unadjusted estimates and, if applicable, confounder-adjusted estimates and their precision (eg, 95% confidence interval). Make clear which confounders were adjusted for and why they were included	11
		(*b*) Report category boundaries when continuous variables were categorized	19
		(*c*) If relevant, consider translating estimates of relative risk into absolute risk for a meaningful time period	Not relevant
Other analyses	17	Report other analyses done—eg analyses of subgroups and interactions, and sensitivity analyses	11-12
**Discussion**			
Key results	18	Summarise key results with reference to study objectives	12
Limitations	19	Discuss limitations of the study, taking into account sources of potential bias or imprecision. Discuss both direction and magnitude of any potential bias	14-15
Interpretation	20	Give a cautious overall interpretation of results considering objectives, limitations, multiplicity of analyses, results from similar studies, and other relevant evidence	12-13
Generalisability	21	Discuss the generalisability (external validity) of the study results	14
**Other information**			
Funding	22	Give the source of funding and the role of the funders for the present study and, if applicable, for the original study on which the present article is based	15

*Give information separately for cases and controls in case-control studies and, if applicable, for exposed and unexposed groups in cohort and cross-sectional studies.

Note: An Explanation and Elaboration article discusses each checklist item and gives methodological background and published examples of transparent reporting. The STROBE checklist is best used in conjunction with this article (freely available on the Web sites of PLoS Medicine at http://www.plosmedicine.org/, Annals of Internal Medicine at http://www.annals.org/, and Epidemiology at http://www.epidem.com/). Information on the STROBE Initiative is available at www.strobe-statement.org.

For peer review only

For peer review only - http://bmjopen.bmj.com/site/about/guidelines.xhtml

Risk of autism spectrum disorder in offspring following paternal use of selective serotonin reuptake inhibitors before conception: a population-based cohort study

Journal:	*BMJ Open*
Manuscript ID	bmjopen-2017-016368.R2
Article Type:	Research
Date Submitted by the Author:	13-Oct-2017
Complete List of Authors:	Yang, Fen; Shanghai Institute of Planned Parenthood Research, Department of Reproductive Epidemiology and Social Science; Aarhus Universitetshospital, Department of Clinical Epidemiology<br>Chen, Jianping; Shanghai Institute of Planned Parenthood Research, Department of Reproductive Epidemiology and Social Science<br>Miao, Mao-Hua; Shanghai Institute of Planned Parenthood Research, Department of Reproductive Epidemiology and Social Science, National Population and Family Planning Key Laboratory of Contraceptive Drugs and Devices<br>Yuan, Wei; Shanghai Institute of Planned Parenthood Research, Reproductive Epidemiology, National Population and Family Planning Key Laboratory of Contraceptive Drugs and Devices<br>Li, Lin; Shanghai Institute of Planned Parenthood Research<br>Liang, Hong; Shanghai Institute of Planned Parenthood Research, Department of Reproductive Epidemiology and Social Science, National Population and Family Planning Key Laboratory of Contraceptive Drugs and Devices<br>Ehrenstein, Vera; Aarhus Universitetshospital<br>Li, Jiong; Aarhus Universitetshospital, Department of Clinical Epidemiology
**Primary Subject Heading**:	Public health
Secondary Subject Heading:	Mental health
Keywords:	MENTAL HEALTH, Child & adolescent psychiatry < PSYCHIATRY, Adult psychiatry < PSYCHIATRY, NEUROPATHOLOGY

Risk of autism spectrum disorder in offspring following paternal use of selective serotonin reuptake inhibitors before conception: a population-based cohort study

Fen Yang[1,2]; Jianping Chen[1]; Maohua Miao[1]; Wei Yuan [1]; Lin Li [1]; Hong Liang[1]; Vera Ehrenstein[2]; Jiong Li [2]

Author Affiliation

[1]Key Lab. of Reproduction Regulation of NPFPC, SIPPR, IRD, Fudan University, Shanghai, 200032, China

[2]Department of Clinical Epidemiology, Aarhus University Hospital, Olof Palmes Alle 43-45, DK-8200 Aarhus N, Denmark

Corresponding Author

Hong Liang, MD, PhD

Key Lab. of Reproduction Regulation of NPFPC, SIPPR, IRD, Fudan University, #779 Lao Hu Min Road, Shanghai, 200032, China.

Email: lucylhcn@163.com;

Phone number: 86 -21-64771683

For peer review only

For peer review only - http://bmjopen.bmj.com/site/about/guidelines.xhtml

Abstract

Objective

The present study aimed to examine the association between paternal selective serotonin reuptake inhibitors (SSRIs) use before conception and the risk of autism spectrum disorder (ASD) in offspring.

Design

A population-based cohort study

Methods

Based on Danish national registers, we conducted a cohort study of 669,922 children born from 1998 to 2008, with follow-up throughout 2013. The children whose fathers used SSRIs during the last 3 months prior to conception were identified as the exposed. Cox regression model was used to estimate the hazard ratio (HR) for ASD in children.

Results

Compared with unexposed children, the exposed had a 1.43-fold higher risk of ASD (HR=1.43, 95% confidence interval [CI]: 1.18-1.74) after adjusting for potential confounders. When extending the exposure window to 1 year before conception, the adjusted HR (aHR) of ASD in children of fathers who used SSRIs only from the last year till the last 3 months prior to conception and who used SSRIs both before and during the last 3 months prior to conception was 1.54 (95%CI: 1.21-1.94) and 1.32 (95%CI: 1.02-1.72), respectively. Whereas, the risk in children of fathers who used SRRIs only during the last 3 months prior to conception decreased and became

For peer review only - http://bmjopen.bmj.com/site/about/guidelines.xhtml

nonsignificant (aHR=1.17, 95%CI: 0.75-1.82).

Conclusions

The increased risk of ASD in the offspring associated with paternal SSRIs use before conception may be attributable to the underlying indications.

Strengths and limitations of this study

- This is the very first study that investigates the link between paternal antidepressants use and ASD risk in children.
- A number of potential confounders, including sociodemographic factors as well as parental psychiatric history, can be adjusted for based on the availability of national health registry data.
- Actual use of SSRIs by fathers during the time period of interest may not be validated.
- Those children who received the diagnosis of ASD from private psychiatrists or remained undiagnosed at the end of the follow-up may not be identified as ASD case.

For peer review only

Introduction

Depression is a common mental health problem that affects approximately 350 million people worldwide[1]. Selective serotonin reuptake inhibitors (SSRIs) are commonly prescribed for the treatment of depression and other anxiety-related disorders. However, an increasing body of evidence suggests that prenatal exposure to SSRIs is associated with adverse obstetrical and neonatal outcomes[2-5]. In addition, recent studies have indicated a possible link between prenatal SSRIs exposure and neurobehavioral problems in children[2, 6-10], which may be due to the biological mechanisms (i.e. dysfunctional serotonin signaling)[11]. Whereas several studies have suggested that the previously observed association between prenatal SSRIs exposure and neurodevelopmental diseases might due to confounding by related indications[12-15].

Autism spectrum disorder (ASD) is one of the major neurodevelopmental disorders characterized by impairments in reciprocal social interaction, communication, and repetitive or stereotypic behavior[16]. The estimated prevalence of ASD has dramatically increased from less than 1 in 2,000 children in 1960s to approximately 1 in 145 children today[17, 18]. Both genetic and environmental risk factors may contribute to ASD [19]. Recently, maternal use of SSRIs during pregnancy has been reported to be associated with an increased risk of ASD in children[10, 20-24].

If maternal SSRIs exposure during pregnancy plays a role in fetal, even in child's, development, an extended effect of earlier exposure on gamete, particularly human sperm, is biologically plausible. Therefore, the potential role of paternal SSRIs use in

For peer review only - http://bmjopen.bmj.com/site/about/guidelines.xhtml

ASD should also be taken into consideration. Studies in the Nordic countries show that one-third of the fathers used prescription drugs during the last 6 months prior to conception [25] , and approximately 1.4% of fathers were dispensed SSRIs during the last 3 months prior to conception [26, 27]. Although the information regarding the potential effects of SSRI use by fathers is limited, the hypothesis of an increased risk of adverse pregnancy or neonatal outcome associated with paternal drug exposure is not new. Several human studies have indicated the adverse effects of paternal SSRIs use, including impaired semen quality and abnormal sperm DNA fragmentation, which has been reported to be associated with diminished fertility, adverse pregnancy outcomes (like pregnancy loss), and an increased risk of childhood disease[28-30].

Little is known on whether paternal SSRIs use before conception contributes to the risk of ASD in offspring. We conducted a population-based cohort study to examine the association between paternal SSRIs use during the last 3 months prior to conception and risk of ASD in offspring, utilizing data from national Danish health registries.

Methods

Study Population

This study was based on several national registers in Denmark. Each Danish resident is assigned a unique personal identification number (a 10-digit civil registration system number used in all registries), which enables accurate linkage of national registries at the individual level[31]. The Danish Medical Birth Registry (DMBR) contains records of all deliveries in Denmark since 1973 and includes information

For peer review only - http://bmjopen.bmj.com/site/about/guidelines.xhtml

about gestational age at birth from 1978[32].Using the DMBR data, we identified a cohort of all singletons born alive in Denmark during the period of January 1, 1998 through December 31, 2008 (n=687,580). We excluded children without linkage to their father (n=16,955) or their mother (n=74), with missing parity (n=208), and with missing or extreme values of gestational age (≤23 weeks or ≥45 weeks, n=421). A total of 669,922 children were included in the analysis.

Data on SSRIs use

Information on SSRIs use was drawn from the Danish National Prescription Registry (DNPR)[33]. Since 1995, this registry has recorded all redeemed prescriptions in Denmark with the following information: the civil registration number of the patient, the dispensing date, the medication code (the WHO Anatomical Therapeutic Chemical (ATC) classification system), the number of packages prescribed, and the number of doses units in package. Selective serotonin reuptake inhibitors use was identified based on ATC codes: fluoxetine (N06AB03), citalopram (N06AB04), paroxetine (N06AB05), sertraline (N06AB06), fluvoxamine (N06AB08), and escitalopram (N06AB10). It is estimated that spermatogenesis takes approximately 74 days[34], therefore we chose the last 3 months prior to conception as the exposure window to cover the susceptible time period. A child was considered exposed if the dispensing date fell within the specified exposure window or the number of days for which the SSRIs medication was supplied overlapped any portion of the exposure window. Children born to fathers who had no prescriptions for SSRIs and no supply overlap during the entire exposure window were considered unexposed. The date of

For peer review only - http://bmjopen.bmj.com/site/about/guidelines.xhtml

conception was estimated by subtracting gestational age from the date of delivery. Data on paternal SSRIs use during the last two years prior to the conception were extracted for further analyses. We also retrieved the information about maternal SSRIs use during the pregnancy.

Autism spectrum disorders

Autism spectrum disorders in children were identified by using the Danish Psychiatric Central Research Register (DPCRR) and the Danish National Patient Register (DNPR). The DPCRR contains diagnostic information on every admission from psychiatric hospitals and psychiatric wards in general hospitals in Denmark since 1969, and includes data on all outpatient visits and emergency room contacts since 1995[35]. The DNPR has collected data on all inpatients from all somatic hospitals in Denmark since 1977 and outpatients from 1995[36]. The combined data from the two registries were used to identify all children diagnosed with ASD. During the study period, the diagnosis of ASD was based on the International Classification of Diseases, 10th version (ICD-10) codes of F84.0 (Childhood autism), F84.1 (Atypical autism), F84.5 (Asperger syndrome), F84.8 (other pervasive developmental disorders) and F84.9 (unspecified pervasive developmental disorders). The quality of the childhood autism diagnosis has been validated and the diagnoses could be verified in 94% of the children with a record in the DPCRR[37]. Children were followed up from birth till first diagnosis of ASD, death, emigration, or December 31, 2013, whichever came first.

Covariates

For peer review only - http://bmjopen.bmj.com/site/about/guidelines.xhtml

Using the Danish nationwide health registers, we retrieved data on characteristics that may be associated with offspring ASD or paternal SSRIs use. For each child, we obtained information on calendar year of birth, gender of the child, birth weight, Apgar score at 5 minutes, gestational age, maternal parity, maternal age at child birth, paternal age at child birth and maternal smoking status during pregnancy from the DMBR. Parents' psychiatric history prior to birth of the index child was obtained from DPCRR by ICD-8 codes 290-315 from 1977 to 1993 and ICD-10 codes F00-F99 from 1994 onward. Furthermore, we identified parents diagnosed with affective disorders before birth of child (specifically, ICD-8 codes 296.09, 296.19, 296.29, 296.39, 296.99, 298.09, 298.19, 300.49, and 301.19, and ICD-10 codes F30–F34 and F38–F39) using the DPCRR.

Statistical Analysis

Cox proportional-hazards regression models (using child's calendar age in years as the underlying timescale) were used to estimate the hazard ratios (HRs) and 95% confidence intervals (CIs) of ASD in children following exposure to paternal SSRIs use before conception. Observations were censored if the child died or emigrated before the end of follow-up.

We adjusted for variables as following: the calendar year of birth (1998-2000, 2001-2003, 2004-2006, 2007-2008), gender of the child (boy, girl), parity (1, 2, ≥3), parental age at child birth (≤25, 26-30, 31-35, and >35 years), maternal smoking status during pregnancy (yes or no), maternal history of psychiatric disorders before birth of child (yes or no), maternal antidepressants use during pregnancy (yes or no)

For peer review only - http://bmjopen.bmj.com/site/about/guidelines.xhtml

in model 1. We additionally adjusted for paternal history of psychiatric disorders before birth of child (yes or no) in model 2. Models were also run with the exclusion of those children with missing data for covariates. The proportional hazard assumption was evaluated for all variables included in the adjusted model by comparing estimated log-minus-log survival curves.

To distinguish the direct effects of SSRIs use from the effects of the indication of SSRIs use, we extended the exposure window to 1 year before conception. We then re-categorized the exposed children into three subgroups: children of fathers who used SSRIs 1) only from the last year till the last 3 months prior to conception (former users); 2) only during the last 3 months prior to conception (current short-term users, hereinafter referred to as 'current users'); 3) both before and during the last 3 months prior to conception (both former and current users). The reference group consisted of those children born to fathers who never used SSRIs medication through the last year before conception.

The stratified analysis was performed to examine whether the association between paternal SSRIs use and ASD in children differed by gender. We also restricted the analyses to children whose mothers neither received antidepressant medication during pregnancy nor had affective disorder before birth of child. To further distinguish the effect of SSRIs medication from that of the main indication (i.e., affective disorders) for SSRIs treatment, we performed stratified analyses according to paternal history of affective disorder before birth of the index child. As for those children born to fathers with affective disorders, the ASD risk we examined could be solely attributable to

For peer review only - http://bmjopen.bmj.com/site/about/guidelines.xhtml

paternal SSRIs use since both of the exposed children and the reference children were with paternal affective disorders.

To control for unmeasured family-related confounding factors (such as genetic liability for neuropsychiatric conditions and early postnatal environmental influences), we conducted sibling-matched analyses by only including the families with exposure-discordant siblings, in which there was at least one child with paternal SSRIs preconception exposure and one child without exposure. Using the stratified Cox proportional-hazards regression with a separate stratum for each family, we estimated the association between paternal SSRIs use before conception and ASD in matched sets of exposure-discordant siblings. In the stratified Cox regression model, we also adjusted for those covariates that varied among siblings with the same father (including parental age at conception, maternal parity, smoking, and maternal AD use during pregnancy).

All analyses were performed using SAS version 9.1 (SAS Institute Inc, Cary, North Carolina, USA).

Results

Among the 669,922 singletons included in the study, 6,870 (1.03%) children were born to fathers who had redeemed a prescription for SSRIs during the last 3 months prior to conception. During the study period, a total of 7,577 children were diagnosed with ASD. The median age at diagnosis of ASD was 6.77 years (interquartile range: 4.84 to 9.48 years). Characteristics of the study population are shown in Table 1. Compared with the unexposed group, there were a higher proportion of exposed

For peer review only - http://bmjopen.bmj.com/site/about/guidelines.xhtml

children born in later calendar years. Fathers in exposed group were more likely to be older at child birth and to have a history of psychiatric disorder (including affective disorder) before birth of child. Mothers of exposed children were characterized as having higher parity, being more likely to be older at child birth, to use antidepressants during pregnancy, and to have a history of psychiatric disorder before birth of child.

Among 61,555 person-years of follow-up, we identified 104 cases of ASD in children born to fathers who used SSRIs during the last 3 months prior to conception (incidence rate: 169 per 100,000 person-years). This incidence rate corresponded to a 62% increased risk of ASD compared with the unexposed group (Table 2). The adjusted HR (aHR) of ASD was 1.54 (95% CI: 1.27-1.88) after adjusting for potential confounders in model 1. After paternal psychiatric history was further adjusted for in model 2, the estimate was 1.43 (95%CI: 1.18-1.74). When extending the exposure window to the last one year prior to conception, after the full adjustment, the HR for ASD in children of fathers who were former users only and who were both former and current users was 1.54 (95%CI: 1.21-1.94) and 1.32 (95%CI: 1.02-1.72), respectively. However, the increased risk decreased and became nonsignificant among children of fathers who were only current users (aHR=1.17, 95%CI: 0.75-1.82, model 2, table2).

The risk estimates of ASD were similar for both boys and girls, regardless of the exposure (Table S1). When we restricted analyses to children whose mothers neither

For peer review only - http://bmjopen.bmj.com/site/about/guidelines.xhtml

used SSRIs during pregnancy nor had affective disorder before birth of child, the results did not change essentially (Table S2).

When the analyses were stratified by paternal affective disorder before birth of the index child, in children born to fathers with a history of affective disorder, there was no association between paternal SSRIs use before conception and ASD in the offspring (model 2: aHR=1.10, 95%CI: 0.61-1.98). For children whose father without affective disorder, the patterns of associations remained similar to those of the main analyses (Table 3).

In the sibling analysis, we identified 5,479 families with more than one child and with at least one child with paternal SSRIs use before conception (Table 4). The risk of ASD in exposed children was decreased when compared with their unexposed siblings (aHR=0.74, 95%CI:0.34-1.59), although with wider confidence interval.

Discussion

In this large population-based cohort study, we observed an increased risk of ASD in the offspring following paternal use of SSRIs during the last 3 months prior to conception. However, the risk attenuated after adjusting for a number of potential confounders, especially fathers' psychiatric conditions. When extending the exposure window to one year before conception, the ASD risk persisted among children born to former users but not current users. In addition, among children born to father with affective disorder, no association was observed. Finally, we performed a sibling analysis which allowed for better control of unmeasured familial confounding and the decreased ASD risk was found among exposed children rather than their unexposed

For peer review only - http://bmjopen.bmj.com/site/about/guidelines.xhtml

siblings. Taken as a whole, our results did not support that paternal SSRIs use before conception could increase the risk of ASD in children.

Recently, concerns have been raised regarding the risk of ASD in the offspring associated with prenatal exposure to SSRIs. Four previous human studies have suggested that in utero exposure to antidepressants would increase the risk of ASD in children[21-24], although another two studies using the Danish registers have reported no significant association [38, 39]. Basic neuro-biologic studies have showed that prenatal SSRIs administration may be part of a causal pathway to ASD by operating directly on the developing brain[40, 41]. Considering the detrimental effect of SSRIs on sperm [28, 29, 42], paternal SSRIs use before conception may also cause adverse effects on pregnancy outcomes. However, till now, only one study have suggested that SSRIs administration to male rats could induce deterioration in fertility and fetal outcomes (including weight gain, organ weights and feed consumption)[43]. To our knowledge, our study is the first to investigate the link between paternal antidepressants use before conception and ASD risk in children. Considering the limited number of ASD cases and the challenges posed by confounding, further studies are needed to corroborate the findings.

In the present study, the increased risk of ASD observed in former users but not in current users implied that indications for paternal SSRIs use may account for the observed association between paternal SSRIs use and ASD in children, which was supported by previous studies suggesting that parental depression and other psychiatric disorders might be associated with the risk of autism in children[44-46].

For peer review only - http://bmjopen.bmj.com/site/about/guidelines.xhtml

Therefore, similar to those studies which focused on the effect of maternal antidepressants use during pregnancy, confounding by indication poses the main challenge in our study. We adopted several analytic strategies to account for such confounding by indication: (1) regression adjustment for paternal psychiatric disorders; (2) negative controls (i.e., former-users analyses); (3) stratified analyses according to paternal history of affective disorders; and (4) sibling analyses. The results of these analyses suggested that paternal psychiatric illness rather than SSRIs exposure might be associated with ASD liability. It was worth noting that, in children born to fathers without affective disorders, there was a significantly increased risk associated with paternal SSRIs use. Most fathers might receive SSRIs treatment from their general practitioner and were therefore not registered with a diagnosis of affective disorders in the hospital system. Therefore, it was possible that the increased risk associated with prenatal SSRIs use was partly confounded by paternal affective disorders diagnosed outside a hospital department for which we were not able to adjust. In addition, other psychiatric diseases related to SSRIs use might also contribute to the observed association.

Our study has several methodological strengths. One strength was that the linkage of several nationwide health registries in Denmark enabled us to conduct a large cohort study with virtually complete follow-up. The definition on exposure to SSRIs was based on a national registry, which eliminated the risk of recall bias caused by self-report. Another strength was that the information on ASD diagnosis was obtained independently of exposure measurement, which could also mitigate the information

For peer review only

For peer review only - http://bmjopen.bmj.com/site/about/guidelines.xhtml

bias. Furthermore, the availability of health registry data enabled us to adjust for a number of potential confounders including sociodemographic factors as well as parental psychiatric history. Besides, we have taken the potential effect of maternal antidepressants use during pregnancy as well as maternal mental disease into consideration. To remove the confounding factors attributing to mothers, we adjusted the maternal SSRIs use in regression model, and also restricted the analyses to children whose mothers neither received antidepressant medication during pregnancy nor had affective disorders before child birth.

Limitations need to be considered when interpreting the results of our study. Firstly, we were unable to validate actual use of SSRIs by fathers during the time period of interest because we relied on medical records of dispensed prescriptions. This may lead to misclassification of exposure status because some people may not take the medication or may take it later. Nevertheless, the misclassification was most likely non-differential, which could bias the association toward null. Besides, some patients may receive SSRIs treatment during inpatient admissions which are not included in the prescription registry. We expect this problem to be minor since those inpatients usually have severe psychiatric disorders and are more likely to continue treatment after discharge. Secondly, the 3-month cut-off point was set based on the fact that spermatogenesis takes approximately 70-90 days in humans. Whereas it may be possible that SSRIs drugs induce sperm damage at the very primitive stage. However, the results did not change markedly after extending the putative exposure period to the last 6 months prior to conception (data not shown). Thirdly, ASD in children was

For peer review only - http://bmjopen.bmj.com/site/about/guidelines.xhtml

ascertained through the DNPR and the DPCRR, which did not include those children who received the diagnosis of ASD from private psychiatrists or remained undiagnosed at the end of the follow-up. However, the prevalence of ASD in our cohort was 1.13%, which was similar to that reported in the United States during the study period (1.14%) [47]. Hence the bias introduced by case identification was expected to be minimal. Fourthly, the age of ASD diagnosis which was used as time event in Cox regression models might be affected by external and extraneous factors. If these factors are differentially distributed in exposed and unexposed group, the actual associations may be biased. We have adjusted for some factors which may influence age of diagnosis to reduce the bias to some extent. However, we could not rule out the confounding effects of unmeasured factors, which is a limitation of the study.

Conclusions

In the study, paternal SSRI use before conception was associated with an increased risk of ASD in the offspring, especially in the former users who took SSRIs over the longer term. However, null association were observed in exposed children with paternal affective disorders, and similar ASD risk were observed among exposed and unexposed siblings, which implicates that paternal underlying indications related to SSRIs use or other unmeasured confounding factors may explain the increased risk.

Footnotes

This cohort study was approved by the Danish Data Protection Agency (Document No. 2013-41-2569).

For peer review only - http://bmjopen.bmj.com/site/about/guidelines.xhtml

Contributors: HL had full access to all the data in the study and took responsibility for the integrity of the data and the accuracy of the data analysis. HL, WY and JL conceptualized and designed the study, and MHM and VE helped with its development. FY, LL and JPC conducted the statistical analysis. HL, JPC and FY interpreted the results and FY drafted the initial manuscript. All authors reviewed the manuscript and approved the final version as submitted.

Funding: The study was supported by the National Natural Science Foundation of China (81428011), the National Key Research and Development Program of China (2016YFC1000505), the European Research Council (ERC-2010-StG-260242-PROGEURO), Nordic Cancer Union (176673,186200), Danish Council for Independent Research (DFF-6110-00019), Karen Elise Jensens Fond (2016), and the Program for Clinical Research Infrastructure (PROCRIN) established by the Lundbeck Foundation and the Novo Nordisk Foundation.

Competing interests: The authors declare that they have no conflict of interest.

Statement of the independence of researchers from funders: The funding sources had no role in the design and conduct of the study; collection, management, analysis, and interpretation of the data; and preparation, review, or approval of the manuscript; and decision to submit the manuscript for publication. The researchers were independent from the funders.

Ethical approval: The study was based on secondary data. No individuals were approached as a result of the study, nor did we access any other data from the participants. This study was approved by the Danish Data Protection Agency

For peer review only - http://bmjopen.bmj.com/site/about/guidelines.xhtml

(Document No. 2013-41-2569). All procedures performed in the study involving human participants were in accordance with the ethical standards of the institutional and/or national research committee and with the 1964 Helsinki declaration and its later amendments or comparable ethical standards. Approval by an institutional review board and informed consent are not required for registry-based research in Denmark.

Data sharing statement: No additional data are available.

Reference

1. Organization WH. Depression [fact sheet no. 369]. 2012. As of September. 2015;30.
2. Alwan S, Friedman JM, Chambers C. Safety of selective serotonin reuptake inhibitors in pregnancy: a review of current evidence. CNS drugs. 2016;30(6):499-515.
3. Wen SW, Yang Q, Garner P, et al. Selective serotonin reuptake inhibitors and adverse pregnancy outcomes. American journal of obstetrics and gynecology. 2006;194(4):961-966.
4. Lund N, Pedersen LH, Henriksen TB. Selective serotonin reuptake inhibitor exposure in utero and pregnancy outcomes. Archives of pediatrics & adolescent medicine. 2009;163(10):949-954.
5. Pedersen LH, Henriksen TB, Vestergaard M, et al. Selective serotonin reuptake inhibitors in pregnancy and congenital malformations: population based cohort study. Bmj. 2009;339:b3569.
6. Oberlander TF, Reebye P, Misri S, et al. Externalizing and attentional behaviors in children of depressed mothers treated with a selective serotonin reuptake inhibitor antidepressant during pregnancy. Archives of pediatrics & adolescent medicine. 2007;161(1):22-29.
7. Nulman I, Koren G, Rovet J, et al. Neurodevelopment of children following prenatal exposure to venlafaxine, selective serotonin reuptake inhibitors, or untreated maternal depression. American Journal of Psychiatry. 2012;169(11):1165-1174.
8. Healy D, Le Noury J, Mangin D. Links between serotonin reuptake inhibition during pregnancy and neurodevelopmental delay/spectrum disorders: A systematic review of epidemiological and physiological evidence. International Journal of Risk & Safety in Medicine. 2016;28(3):125-141.
9. Clements CC, Castro VM, Blumenthal SR, et al. Prenatal antidepressant exposure is associated with risk for attention-deficit hyperactivity disorder but not autism spectrum disorder in a large health system. Molecular psychiatry. 2015;20(6):727-734.
10. Boukhris T, Sheehy O, Mottron L, et al. Antidepressant use during pregnancy and the risk of autism spectrum disorder in children. JAMA pediatrics. 2016;170(2):117-124.
11. Hadjikhani N. Serotonin, pregnancy and increased autism prevalence: is there a link? Medical Hypotheses. 2010;74(5):880-883.
12. Brown HK, Ray JG, Wilton AS, et al. Association Between Serotonergic Antidepressant Use During Pregnancy and Autism Spectrum Disorder in Children. Jama. 2017;317(15):1544-1552.
13. Sujan AC, Rickert ME, Öberg AS, et al. Associations of Maternal Antidepressant Use During the

For peer review only - http://bmjopen.bmj.com/site/about/guidelines.xhtml

First Trimester of Pregnancy With Preterm Birth, Small for Gestational Age, Autism Spectrum Disorder, and Attention-Deficit/Hyperactivity Disorder in Offspring. Jama. 2017;317(15):1553-1562.
14. Oberlander TF, Zwaigenbaum L. Disentangling Maternal Depression and Antidepressant Use During Pregnancy as Risks for Autism in Children. Jama. 2017;317(15):1533-1534.
15. Mezzacappa A, Lasica P-A, Gianfagna F, et al. Risk for Autism Spectrum Disorders According to Period of Prenatal Antidepressant Exposure: A Systematic Review and Meta-analysis. JAMA pediatrics. 2017.
16. Baird G, Simonoff E, Pickles A, et al. Prevalence of disorders of the autism spectrum in a population cohort of children in South Thames: the Special Needs and Autism Project (SNAP). The lancet. 2006;368(9531):210-215.
17. Rutter M. Incidence of autism spectrum disorders: Changes over time and their meaning*. Acta paediatrica. 2005;94(1):2-15.
18. Hill AP, Zuckerman K, Fombonne E. Epidemiology of autism spectrum disorders. Translational Approaches to Autism Spectrum Disorder: Springer 2015:13-38.
19. Newschaffer CJ, Croen LA, Daniels J, et al. The epidemiology of autism spectrum disorders*. Annu Rev Public Health. 2007;28:235-258.
20. Brown HK, Hussain-Shamsy N, Lunsky Y, et al. The association between antenatal exposure to selective serotonin reuptake inhibitors and autism: a systematic review and meta-analysis. The Journal of clinical psychiatry. 2017;78(1):e48.
21. Gidaya NB, Lee BK, Burstyn I, et al. In utero exposure to selective serotonin reuptake inhibitors and risk for autism spectrum disorder. Journal of autism and developmental disorders. 2014;44(10):2558-2567.
22. Croen LA, Grether JK, Yoshida CK, et al. Antidepressant use during pregnancy and childhood autism spectrum disorders. Archives of general psychiatry. 2011;68(11):1104-1112.
23. Rai D, Lee BK, Dalman C, et al. Parental depression, maternal antidepressant use during pregnancy, and risk of autism spectrum disorders: population based case-control study. 2013.
24. Rai D, Lee BK, Dalman C, et al. Antidepressants during pregnancy and autism in offspring: population based cohort study. Bmj. 2017;358:j2811.
25. Schirm E, Pedersen L, Tobi H, et al. Drug use among fathers around time of conception: two register based surveys from Denmark and The Netherlands. Pharmacoepidemiology and drug safety. 2004;13(9):609-613.
26. Engeland A, Bramness JG, Daltveit AK, et al. Prescription drug use among fathers and mothers before and during pregnancy. A population ‐ based cohort study of 106 000 pregnancies in Norway 2004 ‐ 2006. British journal of clinical pharmacology. 2008;65(5):653-660.
27. Crijns I, Bos J, Knol M, et al. Paternal drug use: before and during pregnancy. Expert opinion on drug safety. 2012;11(4):513-518.
28. Tanrikut C, Feldman AS, Altemus M, et al. Adverse effect of paroxetine on sperm. Fertility and sterility. 2010;94(3):1021-1026.
29. Safarinejad MR. Sperm DNA damage and semen quality impairment after treatment with selective serotonin reuptake inhibitors detected using semen analysis and sperm chromatin structure assay. The Journal of urology. 2008;180(5):2124-2128.
30. Lewis S, Aitken R. DNA damage to spermatozoa has impacts on fertilization and pregnancy. Cell and tissue research. 2005;322(1):33-41.
31. Pedersen CB, Gøtzsche H, Møller JØ, et al. The Danish civil registration system. Dan Med Bull.

For peer review only - http://bmjopen.bmj.com/site/about/guidelines.xhtml

2006;53(4):441-449.
32. Knudsen LB, Olsen J. The Danish Medical Birth Registry. Danish medical bulletin. 1998;45(3):320-323.
33. Kildemoes HW, Sørensen HT, Hallas J. The Danish national prescription registry. Scandinavian journal of public health. 2011;39(7 suppl):38-41.
34. Cordier S. Evidence for a role of paternal exposures in developmental toxicity. Basic & clinical pharmacology & toxicology. 2008;102(2):176-181.
35. Mors O, Perto GP, Mortensen PB. The Danish psychiatric central research register. Scandinavian journal of public health. 2011;39(7 suppl):54-57.
36. Andersen TF, Madsen M, Jørgensen J, et al. The Danish National Hospital Register. A valuable source of data for modern health sciences. Danish medical bulletin. 1999;46(3):263-268.
37. Lauritsen MB, Jørgensen M, Madsen KM, et al. Validity of childhood autism in the Danish Psychiatric Central Register: findings from a cohort sample born 1990–1999. Journal of autism and developmental disorders. 2010;40(2):139-148.
38. Christensen J, Parner ET, Vestergaard M. Antidepressant exposure in pregnancy and risk of autism spectrum disorders. Clinical epidemiology. 2013;5:449-459.
39. Hviid A, Melbye M, Pasternak B. Use of selective serotonin reuptake inhibitors during pregnancy and risk of autism. New England Journal of Medicine. 2013;369(25):2406-2415.
40. Maciag D, Simpson KL, Coppinger D, et al. Neonatal antidepressant exposure has lasting effects on behavior and serotonin circuitry. Neuropsychopharmacology. 2006;31(1):47-57.
41. Bonnin A, Levitt P. Fetal, maternal and placental sources of serotonin and new implications for developmental programming of the brain. Neuroscience. 2011;197:1.
42. Tanrikut C, Schlegel PN. Antidepressant-associated changes in semen parameters. Urology. 2007;69(1):185. e185-185. e187.
43. Elmazoudy R, Abdelhameed N, Elmasry A. Paternal dapoxetine administration induced deterioration in reproductive performance, fetal outcome, sexual behavior and biochemistry of male rats. International Journal of Impotence Research. 2015;27(6).
44. Daniels JL, Forssen U, Hultman CM, et al. Parental psychiatric disorders associated with autism spectrum disorders in the offspring. Pediatrics. 2008;121(5):e1357-e1362.
45. Larsson HJ, Eaton WW, Madsen KM, et al. Risk factors for autism: perinatal factors, parental psychiatric history, and socioeconomic status. American journal of epidemiology. 2005;161(10):916-925.
46. Jokiranta E, Brown AS, Heinimaa M, et al. Parental psychiatric disorders and autism spectrum disorders. Psychiatry research. 2013;207(3):203-211.
47. Kogan MD, Blumberg SJ, Schieve LA, et al. Prevalence of parent-reported diagnosis of autism spectrum disorder among children in the US, 2007. Pediatrics. 2009;124(5):1395-1403.

For peer review only - http://bmjopen.bmj.com/site/about/guidelines.xhtml

Table1. Baseline Characteristics of the study population

Characteristic	Paternal SSRIs use during the last 3 months prior to conception (N=6,870)	No paternal SSRIs use during the last 3 months prior to conception (N=663, 052)
**Calendar Year of birth, No. (%)**		
1998-2000	1,021(14.9)	185,784(28.0)
2001-2003	1,526(22.2)	179,952(27.1)
2004-2006	2,305(33.5)	178,664(27.0)
2007-2008	2,018(29.4)	118,652(17.9)
**Gender, No. (%)**		
Boy	3,549(51.7)	340,194(51.3)
Girl	3,321(48.3)	322,858(48.7)
**Birth Weight (g), No. (%)**		
<2500	278(4.1)	22,315(3.4)
2500-3250	1,729(25.2)	154,494(23.3)
3250-4000	3,582(52.1)	348,853(52.6)
4000-8000	1,243(18.1)	132,343(19.9)
Unknown	38(0.5)	5,047(0.8)
**Parity, No. (%)**		
1	2,693(39.2)	282,895(42.7)
2	2,433(35.4)	250,496(37.8)
≥3	1,744(25.4)	129,661(19.5)
**Preterm Birth, No. (%)**		
(<37 weeks)		
No	6,489(94.4)	630,851(95.1)
Yes	381(5.6)	32,201(4.9)
**Apgar score at 5 minutes, No. (%)**		
0-7	91(1.3)	8,224(1.2)
8-9	442(6.4)	39,941(6.0)
10	6,269(91.3)	607,634(91.7)
Unknown	68(1.0)	7,253(1.1)
**Maternal age at child birth (years), No. (%)**		
≤25	958(13.9)	99,273(15.0)
26-30	2,238(32.6)	244,419(36.9)
31-35	2,386(34.7)	225,610(34.0)
>35	1,288(18.8)	93,750(14.1)
**Paternal age at child birth (years), No. (%)**		
≤25	364(5.3)	47,490(7.2)
26-30	1,434(20.9)	180,612(27.2)
31-35	2,395(34.8)	242,702(36.6)
>35	2,677(39.0)	192,248(29.0)

For peer review only

For peer review only - http://bmjopen.bmj.com/site/about/guidelines.xhtml

Table1. Baseline Characteristics of the study population (continued)

**Maternal smoking status[a], No. (%)**		
No	4,326(63.0)	477,574(72.0)
Yes	1,334(19.4)	106,233(16.0)
Unknown	1,210(17.6)	79,245(12.0)
**Maternal AD use during pregnancy, No. (%)**		
No	6,548(94.0)	653,496(98.6)
Yes	412(6.0)	9,556(1.4)
**Maternal history of Affective disorder, No. (%)**		
No	6,607(96.2)	654,234(98.7)
Yes	263(3.8)	8,818(1.3)
**Paternal history of Affective disorder, No. (%)**		
No	6,099 (88.8)	659,639(99.5)
Yes	771(11.2)	3,413(0.5)
**Maternal history of psychiatric disorder, No. (%)**		
No	6,175(89.9)	626,840(94.5)
Yes	695(10.1)	36,212(5.5)
**Paternal history of psychiatric disorder, No. (%)**		
No	5,092 (74.1)	630,9909(95.2)
Yes	1,178(25.9)	32,062(4.8)

Abbreviations: SSRI, selective serotonin reuptake inhibitor; N, number; AD, antidepressant drugs;

[a] Maternal smoking information was missing among those children born from 2007 to 2008.

For peer review only - http://bmjopen.bmj.com/site/about/guidelines.xhtml

Table2. Association between paternal SSRIs use before conception and ASD in offspring

Paternal SSRIs use before conception	Offspring with ASD no.	Follow-up no. of person-yr	Hazard Ratio (95% CI)		
			Crude	Model 1[a]	Model 2[b]
No use during the last 3 months prior to conception	7,473	6,765,205	Ref	Ref	Ref
Use during the last 3 months prior to conception	104	61,555	1.62(1.33-1.96)	1.54(1.27-1.88)	1.43(1.18-1.74)
Sub-analysis: Paternal SSRIs use during the last 1 year before conception					
No use during the last 1 year prior to conception	7,429	6,736,654	Ref	Ref	Ref
Use only from the last 1 year to the last 3 months prior to conception	71	39,422	1.71(1.35-2.16)	1.66(1.31-2.09)	1.54(1.21-1.94)
Use only during the last 3 months prior to conception	20	14,738	1.29(0.83-2.00)	1.24 (0.80-1.93)	1.17(0.75-1.82)
Use both before and during the last 3 months prior to conception	57	35,946	1.52(1.17-1.97)	1.43(1.10-1.86)	1.32(1.02-1.72)

Abbreviations: AD, antidepressant drugs; SSRI, selective serotonin reuptake inhibitor; ASD, Autism Spectrum Disorder; No., number; HR, Hazard Ratio

[a] Adjusted for calendar year of birth, sex, parity, mother age, father age, maternal smoking, mother psychiatric history, maternal AD use during pregnancy

[b] Model 1 further adjusted for father psychiatric history

For peer review only - http://bmjopen.bmj.com/site/about/guidelines.xhtml

Table3. Association between paternal SSRIs use before conception and ASD in offspring born to fathers diagnosed with affective disorder or not

Paternal SSRIs use before conception	Offspring with ASD no.	Follow-up no. of person-yr	Hazard Ratio (95% CI)		
			Crude	Model 1[a]	Model 2[b]
**Fathers with affective disorder**					
No use during the last 3 months prior to conception	58	30,908	Ref	Ref	Ref
Use during the last 3 months prior to conception	14	6,625	1.17(0.65-2.09)	1.10(0.61-1.98)	1.10(0.61-1.98)
Sub-analysis: ***Paternal SSRIs use during the last 1 year before conception***					
No use during the last 1 year prior to conception	53	28,033	Ref	Ref	Ref
Use only from the last 1 year to the last 3 months prior to conception	8	4,082	1.07(0.51-2.25)	1.08(0.51-2.30)	1.08(0.51-2.30)
Use only during the last 3 months prior to conception	2	1,164	0.91(0.22-3.75)	0.89(0.21-3.75)	0.89(0.21-3.75)
Use both before and during the last 3 months prior to conception	9	4,254	1.16(0.57-2.35)	1.10(0.55-2.22)	1.10(0.55-2.22)
**Fathers without affective disorder**					
No use during the last 3 months prior to conception	7,415	6,752,899	Ref	Ref	Ref
Use during the last 3 months prior to conception	90	36,329	1.57(1.27-1.93)	1.52(1.23-1.87)	1.45(1.18-1.79)
Sub-analysis: ***Paternal SSRIs use during the last 1 year before conception***					
No use during the last 1 year prior to conception	7,376	6,714,926	Ref	Ref	Ref
Use only from the last 1 year to the last 3 months prior to conception	63	37,973	1.69(1.32-2.17)	1.64(1.28-2.11)	1.57(1.23-2.02)
Use only during the last 3 months prior to conception	18	7,354	1.26(0.79-2.00)	1.23(0.77-1.95)	1.19(0.75-1.88)
Use both before and during the last 3 months prior to conception	48	28,974	1.45(1.09-1.93)	1.38(1.04-1.84)	1.32(0.99-1.76)

Abbreviations: AD, antidepressant drugs; SSRI, selective serotonin reuptake inhibitor; ASD, Autism Spectrum Disorder; No., number; HR, Hazard Ratio

[a] Adjusted for calendar year of birth, parity, mother age, father age, maternal smoking, mother psychiatric history , maternal AD use during pregnancy

[b] Model 1 further adjusted for father psychiatric history

For peer review only - http://bmjopen.bmj.com/site/about/guidelines.xhtml

For peer review only

Table4. Paternal SSRI use before pregnancy and ASD in exposed and unexposed siblings from 5,479 families

Paternal SSRIs use before conception	Number of offspring no.	Offspring with ASD no.	Hazard Ratio (95% CI)	
			Crude	Model 1[a]
No use during the last 3 months prior to conception	2,792	45	Ref	Ref
Use during the last 3 months prior to conception	2,687	23	0.53(0.31-0.91)	0.74(0.34-1.59)

Abbreviations: AD, antidepressant drugs; SSRI, selective serotonin reuptake inhibitor; ASD, Autism Spectrum Disorder; No., number; HR, Hazard Ratio

[a] Adjusted for sex, parity, mother age, father age, maternal smoking, maternal AD use during pregnancy

For peer review only

TablesS1. Association between paternal SSRIs use before conception and ASD in offspring: analyses stratified by gender

Paternal SSRIs use before conception	Offspring with ASD no.	Follow-up no. of person-yr	Hazard Ratio (95% CI)		
			Crude	Model 1[a]	Model 2[b]
**Boys**					
No use during the last 3 months prior to conception	6,005	3,459,592	Ref	Ref	Ref
Use during the last 3 months prior to conception	84	31,642	1.61(1.30-2.00)	1.55(1.25-1.92)	1.44(1.16-1.79)
Sub-analysis: ***Paternal SSRIs use during the last 1 year before conception***					
No use during the last 1 year prior to conception	5,969	3,444,956	Ref	Ref	Ref
Use only from the last 1 year to the last 3 months prior to conception	58	20,031	1.74(1.34-2.25)	1.69(1.30-2.19)	1.58(1.22-2.05)
Use only during the last 3 months prior to conception	18	7,622	1.42(0.89-2.26)	1.39(0.87-2.21)	1.31(0.83-2.09)
Use both before and during the last 3 months prior to conception	44	18,623	1.44 (1.07-1.93)	1.36(1.01-1.84)	1.26(0.94-1.71)
**Girls**					
No use during the last 3 months prior to conception	1,468	3,305,613	Ref	Ref	Ref
Use during the last 3 months prior to conception	20	29,914	1.63(1.05-2.53)	1.54(0.99-2.40)	1.41(0.90-2.20)
Sub-analysis: ***Paternal SSRIs use during the last 1 year before conception***					
No use during the last 1 year prior to conception	1,460	3,291,698	Ref	Ref	Ref
Use only from the last 1 year to the last 3 months prior to conception	13	19,390	1.61(0.93-2.78)	1.53(0.88-2.64)	1.37(0.79-2.38)
Use only during the last 3 months prior to conception	2	7,116	0.67(0.17-2.69)	0.64(0.16-2.55)	0.59(0.15-2.36)
Use both before and during the last 3 months prior to conception	13	75,323	1.84(1.06-3.17)	1.74(1.01-3.01)	1.59(0.92-2.75)

Abbreviations: AD, antidepressant drugs; SSRI, selective serotonin reuptake inhibitor; ASD, Autism Spectrum Disorder; No., number; HR, Hazard Ratio

[a] Adjusted for calendar year of birth, parity, mother age, father age, maternal smoking, mother psychiatric history, maternal AD use during pregnancy

[b] Model 1 further adjusted for father psychiatric history

For peer review only

TableS2. Association between paternal SSRIs use before conception and ASD in offspring born to mother who neither received antidepressants medication during pregnancy nor had affective disorders before birth of child

Paternal SSRIs use before conception	Offspring with ASD no.	Follow-up no. of person-yr	Hazard Ratio (95% CI)		
			Crude	Model 1[a]	Model 2[b]
No use during the last 3 months prior to conception	7,184	6,627,720	Ref	Ref	Ref
Use during the last 3 months prior to conception	91	57,081	1.55(1.26-1.91)	1.57(1.28-1.93)	1.45(1.18-1.79)
Sub-analysis:					
Paternal SSRIs use during the last 1 year before conception					
No use during the last 1 year prior to conception	7,144	6,600,852	Ref	Ref	Ref
Use only from the last 1 year to the last 3 months prior to conception	64	37,032	1.67(1.30-2.13)	1.68(1.31-2.15)	1.56(1.22-1.99)
Use only during the last 3 months prior to conception	16	13,634	1.13(0.69-1.85)	1.15(0.71-1.89)	1.08(0.66-1.77)
Use both before and during the last 3 months prior to conception	51	33,283	1.50(1.13-1.97)	1.50(1.13-1.97)	1.38(1.05-1.82)

Abbreviations: AD, antidepressant drugs; SSRI, selective serotonin reuptake inhibitor; ASD, Autism Spectrum Disorder; No., number; HR, Hazard Ratio

[a] Adjusted for calendar year of birth, parity, mother age, father age, maternal smoking, mother psychiatric history

[b] Model 1 further adjusted for father psychiatric history

For peer review only - http://bmjopen.bmj.com/site/about/guidelines.xhtml

STROBE Statement—checklist of items that should be included in reports of observational studies

	Item No	Recommendation	Page No.
**Title and abstract**	1	(*a*) Indicate the study's design with a commonly used term in the title or the abstract	2
		(*b*) Provide in the abstract an informative and balanced summary of what was done and what was found	2-3
**Introduction**			
Background/rationale	2	Explain the scientific background and rationale for the investigation being reported	4
Objectives	3	State specific objectives, including any prespecified hypotheses	5
**Methods**			
Study design	4	Present key elements of study design early in the paper	6
Setting	5	Describe the setting, locations, and relevant dates, including periods of recruitment, exposure, follow-up, and data collection	6-8
Participants	6	(*a*) *Cohort study*—Give the eligibility criteria, and the sources and methods of selection of participants. Describe methods of follow-up<br>*Case-control study*—Give the eligibility criteria, and the sources and methods of case ascertainment and control selection. Give the rationale for the choice of cases and controls<br>*Cross-sectional study*—Give the eligibility criteria, and the sources and methods of selection of participants	6-8
		(*b*) *Cohort study*—For matched studies, give matching criteria and number of exposed and unexposed<br>*Case-control study*—For matched studies, give matching criteria and the number of controls per case	
Variables	7	Clearly define all outcomes, exposures, predictors, potential confounders, and effect modifiers. Give diagnostic criteria, if applicable	6-8
Data sources/ measurement	8*	For each variable of interest, give sources of data and details of methods of assessment (measurement). Describe comparability of assessment methods if there is more than one group	6-8
Bias	9	Describe any efforts to address potential sources of bias	8-10
Study size	10	Explain how the study size was arrived at	10
Quantitative variables	11	Explain how quantitative variables were handled in the analyses. If applicable, describe which groupings were chosen and why	8-9
Statistical methods	12	(*a*) Describe all statistical methods, including those used to control for confounding	8-10
		(*b*) Describe any methods used to examine subgroups and interactions	9
		(*c*) Explain how missing data were addressed	9
		(*d*) *Cohort study*—If applicable, explain how loss to follow-up was addressed<br>*Case-control study*—If applicable, explain how matching of cases and controls was addressed<br>*Cross-sectional study*—If applicable, describe analytical methods taking account of sampling strategy	Not applicable
		(*e*) Describe any sensitivity analyses	9-10

For peer review only - http://bmjopen.bmj.com/site/about/guidelines.xhtml

**Results**			
Participants	13*	(a) Report numbers of individuals at each stage of study—eg numbers potentially eligible, examined for eligibility, confirmed eligible, included in the study, completing follow-up, and analysed	10
		(b) Give reasons for non-participation at each stage	Not applicable
		(c) Consider use of a flow diagram	
Descriptive data	14*	(a) Give characteristics of study participants (eg demographic, clinical, social) and information on exposures and potential confounders	10
		(b) Indicate number of participants with missing data for each variable of interest	22
		(c) *Cohort study*—Summarise follow-up time (eg, average and total amount)	11
Outcome data	15*	*Cohort study*—Report numbers of outcome events or summary measures over time	10-11
		Case-control study—Report numbers in each exposure category, or summary measures of exposure	
		Cross-sectional study—Report numbers of outcome events or summary measures	
Main results	16	(*a*) Give unadjusted estimates and, if applicable, confounder-adjusted estimates and their precision (eg, 95% confidence interval). Make clear which confounders were adjusted for and why they were included	11
		(*b*) Report category boundaries when continuous variables were categorized	21
		(*c*) If relevant, consider translating estimates of relative risk into absolute risk for a meaningful time period	Not relevant
Other analyses	17	Report other analyses done—eg analyses of subgroups and interactions, and sensitivity analyses	11-12
**Discussion**			
Key results	18	Summarise key results with reference to study objectives	12
Limitations	19	Discuss limitations of the study, taking into account sources of potential bias or imprecision. Discuss both direction and magnitude of any potential bias	15-16
Interpretation	20	Give a cautious overall interpretation of results considering objectives, limitations, multiplicity of analyses, results from similar studies, and other relevant evidence	13-14
Generalisability	21	Discuss the generalisability (external validity) of the study results	14-15
**Other information**			
Funding	22	Give the source of funding and the role of the funders for the present study and, if applicable, for the original study on which the present article is based	17

*Give information separately for cases and controls in case-control studies and, if applicable, for exposed and unexposed groups in cohort and cross-sectional studies.

Note: An Explanation and Elaboration article discusses each checklist item and gives methodological background and published examples of transparent reporting. The STROBE checklist is best used in conjunction with this article (freely available on the Web sites of PLoS Medicine at http://www.plosmedicine.org/, Annals of Internal Medicine at http://www.annals.org/, and Epidemiology at http://www.epidem.com/). Information on the STROBE Initiative is available at www.strobe-statement.org.

For peer review only - http://bmjopen.bmj.com/site/about/guidelines.xhtml

BMJ Open

Risk of autism spectrum disorder in offspring following paternal use of selective serotonin reuptake inhibitors before conception: a population-based cohort study

Journal:	*BMJ Open*
Manuscript ID	bmjopen-2017-016368.R3
Article Type:	Research
Date Submitted by the Author:	10-Nov-2017
Complete List of Authors:	Yang, Fen; Shanghai Institute of Planned Parenthood Research, Department of Reproductive Epidemiology and Social Science; Aarhus Universitetshospital, Department of Clinical Epidemiology<br>Chen, Jianping; Shanghai Institute of Planned Parenthood Research, Department of Reproductive Epidemiology and Social Science<br>Miao, Mao-Hua; Shanghai Institute of Planned Parenthood Research, Department of Reproductive Epidemiology and Social Science, National Population and Family Planning Key Laboratory of Contraceptive Drugs and Devices<br>Yuan, Wei; Shanghai Institute of Planned Parenthood Research, Reproductive Epidemiology, National Population and Family Planning Key Laboratory of Contraceptive Drugs and Devices<br>Li, Lin; Shanghai Institute of Planned Parenthood Research<br>Liang, Hong; Shanghai Institute of Planned Parenthood Research, Department of Reproductive Epidemiology and Social Science, National Population and Family Planning Key Laboratory of Contraceptive Drugs and Devices<br>Ehrenstein, Vera; Aarhus Universitetshospital<br>Li, Jiong; Aarhus Universitetshospital, Department of Clinical Epidemiology
**Primary Subject Heading**:	Public health
Secondary Subject Heading:	Mental health
Keywords:	MENTAL HEALTH, Child & adolescent psychiatry < PSYCHIATRY, Adult psychiatry < PSYCHIATRY, NEUROPATHOLOGY

SCHOLARONE™
Manuscripts

Risk of autism spectrum disorder in offspring following paternal use of selective serotonin reuptake inhibitors before conception: a population-based cohort study

Fen Yang[1,2]; Jianping Chen[1]; Maohua Miao[1]; Wei Yuan [1]; Lin Li [1]; Hong Liang[1]; Vera Ehrenstein[2]; Jiong Li [2]

Author Affiliation

[1]Key Lab. of Reproduction Regulation of NPFPC, SIPPR, IRD, Fudan University, Shanghai, 200032, China

[2]Department of Clinical Epidemiology, Aarhus University Hospital, Olof Palmes Alle 43-45, DK-8200 Aarhus N, Denmark

Corresponding Author

Hong Liang, MD, PhD

Key Lab. of Reproduction Regulation of NPFPC, SIPPR, IRD, Fudan University, #779 Lao Hu Min Road, Shanghai, 200032, China.

Email: lucylhcn@163.com;

Phone number: 86 -21-64771683

For peer review only

For peer review only - http://bmjopen.bmj.com/site/about/guidelines.xhtml

Abstract

Objective

The present study aimed to examine the association between paternal selective serotonin reuptake inhibitors (SSRIs) use before conception and the risk of autism spectrum disorder (ASD) in offspring.

Design

A population-based cohort study

Methods

We conducted a cohort study of 669,922 children born from 1998 to 2008, with follow-up throughout 2013. Based on Danish national registers, we linked information on paternal use of SSRIs, ASD diagnosed in children, and a range of potential confounders. The children whose fathers used SSRIs during the last 3 months prior to conception were identified as the exposed. Cox regression model was used to estimate the hazard ratio (HR) for ASD in children.

Results

Compared with unexposed children, the exposed had a 1.62-fold higher risk of ASD (95% confidence interval [CI]: 1.33-1.96) and the risk attenuated after adjusting for potential confounders, especially fathers' psychiatric conditions (HR=1.43, 95% CI: 1.18-1.74). When extending the exposure window to 1 year before conception, the increased risk persisted in children of fathers using SSRIs only from the last year till the last 3 months prior to conception (HR=1.54, 95%CI: 1.21-1.94) but not in children of fathers using SSRIs only during the last 3 months prior to conception

For peer review only

(HR=1.17, 95%CI: 0.75-1.82). We also performed stratified analyses according to paternal history of affective disorders and observed no increased ASD risk among children whose father had affective disorders. Besides, the sibling analysis showed that the ASD risk did not increase among exposed children compared with their unexposed siblings.

Conclusions

The mildly increased risk of ASD in the offspring associated with paternal SSRIs use before conception may be attributable to paternal underlying psychiatric indications related to SSRIs use or other unmeasured confounding factors.

Strengths and limitations of this study

- This is the very first study that investigates the association between paternal antidepressants use and ASD risk in children.
- A number of potential confounders, including sociodemographic factors as well as parental psychiatric history, can be adjusted for based on the availability of national health registry data.
- Actual use of SSRIs by fathers during the time period of interest may not be validated.
- Those children who received the diagnosis of ASD from private psychiatrists or remained undiagnosed at the end of the follow-up may not be identified as ASD case.

For peer review only - http://bmjopen.bmj.com/site/about/guidelines.xhtml

Introduction

Depression is a common mental health problem that affects approximately 350 million people worldwide[1]. Selective serotonin reuptake inhibitors (SSRIs) are commonly prescribed for the treatment of depression and other anxiety-related disorders. However, an increasing body of evidence suggests that prenatal exposure to SSRIs is associated with adverse obstetrical and neonatal outcomes[2-5]. In addition, recent studies have indicated a possible link between prenatal SSRIs exposure and neurobehavioral problems in children[2, 6-10], which may be due to the biological mechanisms (i.e. dysfunctional serotonin signaling)[11]. Whereas several studies have suggested that the previously observed association between prenatal SSRIs exposure and neurodevelopmental diseases might due to confounding by related indications[12-15].

Autism spectrum disorder (ASD) is one of the major neurodevelopmental disorders characterized by impairments in reciprocal social interaction, communication, and repetitive or stereotypic behavior[16]. The estimated prevalence of ASD has dramatically increased from less than 1 in 2,000 children in 1960s to approximately 1 in 145 children today[17, 18]. Both genetic and environmental risk factors may contribute to ASD [19]. Recently, maternal use of SSRIs during pregnancy has been reported to be associated with an increased risk of ASD in children[10, 20-24].

If maternal SSRIs exposure during pregnancy plays a role in fetal, even in child's, development, an extended effect of earlier exposure on gamete, particularly human sperm, is biologically plausible. Therefore, the potential role of paternal SSRIs use in

For peer review only - http://bmjopen.bmj.com/site/about/guidelines.xhtml

ASD should also be taken into consideration. Studies in the Nordic countries show that one-third of the fathers used prescription drugs during the last 6 months prior to conception [25] , and approximately 1.4% of fathers were dispensed SSRIs during the last 3 months prior to conception [26, 27]. Although the information regarding the potential effects of SSRI use by fathers is limited, the hypothesis of an increased risk of adverse pregnancy or neonatal outcome associated with paternal drug exposure is not new. Several human studies have indicated the adverse effects of paternal SSRIs use, including impaired semen quality and abnormal sperm DNA fragmentation, which has been reported to be associated with diminished fertility, adverse pregnancy outcomes (like pregnancy loss), and an increased risk of childhood disease[28-30].

Little is known on whether paternal SSRIs use before conception contributes to the risk of ASD in offspring. We conducted a population-based cohort study to examine the association between paternal SSRIs use during the last 3 months prior to conception and risk of ASD in offspring, utilizing data from national Danish health registries.

Methods

Study Population

This study was based on several national registers in Denmark. Each Danish resident is assigned a unique personal identification number (a 10-digit civil registration system number used in all registries), which enables accurate linkage of national registries at the individual level[31]. The Danish Medical Birth Registry (DMBR) contains records of all deliveries in Denmark since 1973 and includes information

For peer review only

about gestational age at birth from 1978[32].Using the DMBR data, we identified a cohort of all singletons born alive in Denmark during the period of January 1, 1998 through December 31, 2008 (n=687,580). We excluded children without linkage to their father (n=16,955) or their mother (n=74), with missing parity (n=208), and with missing or extreme values of gestational age (≤23 weeks or ≥45 weeks, n=421). A total of 669,922 children were included in the analysis.

Data on SSRIs use

Information on SSRIs use was drawn from the Danish National Prescription Registry (DNPR)[33]. Since 1995, this registry has recorded all redeemed prescriptions in Denmark with the following information: the civil registration number of the patient, the dispensing date, the medication code (the WHO Anatomical Therapeutic Chemical (ATC) classification system), the number of packages prescribed, and the number of doses units in package. Selective serotonin reuptake inhibitors use was identified based on ATC codes: fluoxetine (N06AB03), citalopram (N06AB04), paroxetine (N06AB05), sertraline (N06AB06), fluvoxamine (N06AB08), and escitalopram (N06AB10). It is estimated that spermatogenesis takes approximately 74 days[34], therefore we chose the last 3 months prior to conception as the exposure window to cover the susceptible time period. A child was considered exposed if the dispensing date fell within the specified exposure window or the number of days for which the SSRIs medication was supplied overlapped any portion of the exposure window. Children born to fathers who had no prescriptions for SSRIs and no supply overlap during the entire exposure window were considered unexposed. The date of

For peer review only

conception was estimated by subtracting gestational age from the date of delivery. Data on paternal SSRIs use during the last two years prior to the conception were extracted for further analyses. We also retrieved the information about maternal SSRIs use during the pregnancy.

Autism spectrum disorders

Autism spectrum disorders in children were identified by using the Danish Psychiatric Central Research Register (DPCRR) and the Danish National Patient Register (DNPR). The DPCRR contains diagnostic information on every admission from psychiatric hospitals and psychiatric wards in general hospitals in Denmark since 1969, and includes data on all outpatient visits and emergency room contacts since 1995[35]. The DNPR has collected data on all inpatients from all somatic hospitals in Denmark since 1977 and outpatients from 1995[36]. The combined data from the two registries were used to identify all children diagnosed with ASD. During the study period, the diagnosis of ASD was based on the International Classification of Diseases, 10th version (ICD-10) codes of F84.0 (Childhood autism), F84.1 (Atypical autism), F84.5 (Asperger syndrome), F84.8 (other pervasive developmental disorders) and F84.9 (unspecified pervasive developmental disorders). The quality of the childhood autism diagnosis has been validated and the diagnoses could be verified in 94% of the children with a record in the DPCRR[37]. Children were followed up from birth till first diagnosis of ASD, death, emigration, or December 31, 2013, whichever came first.

Covariates

For peer review only - http://bmjopen.bmj.com/site/about/guidelines.xhtml

Using the Danish nationwide health registers, we retrieved data on characteristics that may be associated with offspring ASD or paternal SSRIs use. For each child, we obtained information on calendar year of birth, gender of the child, birth weight, Apgar score at 5 minutes, gestational age, maternal parity, maternal age at child birth, paternal age at child birth and maternal smoking status during pregnancy from the DMBR. Parents' psychiatric history prior to birth of the index child was obtained from DPCRR by ICD-8 codes 290-315 from 1977 to 1993 and ICD-10 codes F00-F99 from 1994 onward. Furthermore, we identified parents diagnosed with affective disorders before birth of child (specifically, ICD-8 codes 296.09, 296.19, 296.29, 296.39, 296.99, 298.09, 298.19, 300.49, and 301.19, and ICD-10 codes F30–F34 and F38–F39) using the DPCRR.

Statistical Analysis

Cox proportional-hazards regression models (using child's calendar age in years as the underlying timescale) were used to estimate the hazard ratios (HRs) and 95% confidence intervals (CIs) of ASD in children following exposure to paternal SSRIs use before conception. Observations were censored if the child died or emigrated before the end of follow-up.

We adjusted for variables as following: the calendar year of birth (1998-2000, 2001-2003, 2004-2006, 2007-2008), gender of the child (boy, girl), parity (1, 2, ≥3), parental age at child birth (≤25, 26-30, 31-35, and >35 years), maternal smoking status during pregnancy (yes or no), maternal history of psychiatric disorders before birth of child (yes or no), maternal antidepressants use during pregnancy (yes or no)

For peer review only - http://bmjopen.bmj.com/site/about/guidelines.xhtml

in model 1. We additionally adjusted for paternal history of psychiatric disorders before birth of child (yes or no) in model 2. Models were also run with the exclusion of those children with missing data for covariates. The proportional hazard assumption was evaluated for all variables included in the adjusted model by comparing estimated log-minus-log survival curves.

To distinguish the direct effects of SSRIs use from the effects of the indication of SSRIs use, we extended the exposure window to 1 year before conception. We then re-categorized the exposed children into three subgroups: children of fathers who used SSRIs 1) only from the last year till the last 3 months prior to conception (former users); 2) only during the last 3 months prior to conception (current short-term users, hereinafter referred to as 'current users'); 3) both before and during the last 3 months prior to conception (both former and current users). The reference group consisted of those children born to fathers who never used SSRIs medication through the last year before conception.

The stratified analysis was performed to examine whether the association between paternal SSRIs use and ASD in children differed by gender. We also restricted the analyses to children whose mothers neither received antidepressant medication during pregnancy nor had affective disorder before birth of child. To further distinguish the effect of SSRIs medication from that of the main indication (i.e., affective disorders) for SSRIs treatment, we performed stratified analyses according to paternal history of affective disorder before birth of the index child. As for those children born to fathers with affective disorders, the ASD risk we examined could be solely attributable to

For peer review only - http://bmjopen.bmj.com/site/about/guidelines.xhtml

paternal SSRIs use since both of the exposed children and the reference children were with paternal affective disorders.

To control for unmeasured family-related confounding factors (such as genetic liability for neuropsychiatric conditions and early postnatal environmental influences), we conducted sibling-matched analyses by only including the families with exposure-discordant siblings, in which there was at least one child with paternal SSRIs preconception exposure and one child without exposure. Using the stratified Cox proportional-hazards regression with a separate stratum for each family, we estimated the association between paternal SSRIs use before conception and ASD in matched sets of exposure-discordant siblings. In the stratified Cox regression model, we also adjusted for those covariates that varied among siblings with the same father (including parental age at conception, maternal parity, smoking, and maternal AD use during pregnancy).

All analyses were performed using SAS version 9.1 (SAS Institute Inc, Cary, North Carolina, USA).

Results

Among the 669,922 singletons included in the study, 6,870 (1.03%) children were born to fathers who had redeemed a prescription for SSRIs during the last 3 months prior to conception. During the study period, a total of 7,577 children were diagnosed with ASD. The median age at diagnosis of ASD was 6.77 years (interquartile range: 4.84 to 9.48 years). Characteristics of the study population are shown in Table 1. Compared with the unexposed group, there were a higher proportion of exposed

For peer review only - http://bmjopen.bmj.com/site/about/guidelines.xhtml

children born in later calendar years. Fathers in exposed group were more likely to be older at child birth and to have a history of psychiatric disorder (including affective disorder) before birth of child. Mothers of exposed children were characterized as having higher parity, being more likely to be older at child birth, to use antidepressants during pregnancy, and to have a history of psychiatric disorder before birth of child.

Among 61,555 person-years of follow-up, we identified 104 cases of ASD in children born to fathers who used SSRIs during the last 3 months prior to conception (incidence rate: 169 per 100,000 person-years). This incidence rate corresponded to a 62% increased risk of ASD compared with the unexposed group (Table 2). The adjusted HR (aHR) of ASD was 1.54 (95% CI: 1.27-1.88) after adjusting for potential confounders in model 1. After paternal psychiatric history was further adjusted for in model 2, the estimate was 1.43 (95%CI: 1.18-1.74). When extending the exposure window to the last one year prior to conception, after the full adjustment, the HR for ASD in children of fathers who were former users only and who were both former and current users was 1.54 (95%CI: 1.21-1.94) and 1.32 (95%CI: 1.02-1.72), respectively. However, the increased risk decreased and became nonsignificant among children of fathers who were only current users (aHR=1.17, 95%CI: 0.75-1.82, model 2, table2).

The risk estimates of ASD were similar for both boys and girls, regardless of the exposure (Table S1). When we restricted analyses to children whose mothers neither

For peer review only - http://bmjopen.bmj.com/site/about/guidelines.xhtml

used SSRIs during pregnancy nor had affective disorder before birth of child, the results did not change essentially (Table S2).

When the analyses were stratified by paternal affective disorder before birth of the index child, in children born to fathers with a history of affective disorder, there was no association between paternal SSRIs use before conception and ASD in the offspring (model 2: aHR=1.10, 95%CI: 0.61-1.98). For children whose father without affective disorder, the patterns of associations remained similar to those of the main analyses (Table 3).

In the sibling analysis, we identified 5,479 families with more than one child and with at least one child with paternal SSRIs use before conception (Table 4). The risk of ASD in exposed children was decreased when compared with their unexposed siblings (aHR=0.74, 95%CI:0.34-1.59), although with wider confidence interval.

Discussion

In this large population-based cohort study, we observed an increased risk of ASD in the offspring following paternal use of SSRIs during the last 3 months prior to conception. However, the risk attenuated after adjusting for a number of potential confounders, especially fathers' psychiatric conditions. When extending the exposure window to one year before conception, the ASD risk persisted among children born to former users but not current users. In addition, among children born to father with affective disorder, no association was observed. Finally, we performed a sibling analysis which allowed for better control of unmeasured familial confounding and the decreased ASD risk was found among exposed children rather than their unexposed

For peer review only - http://bmjopen.bmj.com/site/about/guidelines.xhtml

siblings. Taken as a whole, our results did not support that paternal SSRIs use before conception could increase the risk of ASD in children.

Recently, concerns have been raised regarding the risk of ASD in the offspring associated with prenatal exposure to SSRIs. Four previous human studies have suggested that in utero exposure to antidepressants would increase the risk of ASD in children[21-24], although another two studies using the Danish registers have reported no significant association [38, 39]. Basic neuro-biologic studies have showed that prenatal SSRIs administration may be part of a causal pathway to ASD by operating directly on the developing brain[40, 41]. Considering the detrimental effect of SSRIs on sperm [28, 29, 42], paternal SSRIs use before conception may also cause adverse effects on pregnancy outcomes. However, till now, only one study have suggested that SSRIs administration to male rats could induce deterioration in fertility and fetal outcomes (including weight gain, organ weights and feed consumption)[43]. To our knowledge, our study is the first to investigate the link between paternal antidepressants use before conception and ASD risk in children. Considering the limited number of ASD cases and the challenges posed by confounding, further studies are needed to corroborate the findings.

In the present study, the increased risk of ASD observed in former users but not in current users implied that indications for paternal SSRIs use may account for the observed association between paternal SSRIs use and ASD in children, which was supported by previous studies suggesting that parental depression and other psychiatric disorders might be associated with the risk of autism in children[44-46].

For peer review only - http://bmjopen.bmj.com/site/about/guidelines.xhtml

Therefore, similar to those studies which focused on the effect of maternal antidepressants use during pregnancy, confounding by indication poses the main challenge in our study. We adopted several analytic strategies to account for such confounding by indication: (1) regression adjustment for paternal psychiatric disorders; (2) negative controls (i.e., former-users analyses); (3) stratified analyses according to paternal history of affective disorders; and (4) sibling analyses. The results of these analyses suggested that paternal psychiatric illness rather than SSRIs exposure might be associated with ASD liability. It was worth noting that, in children born to fathers without affective disorders, there was a significantly increased risk associated with paternal SSRIs use. Most fathers might receive SSRIs treatment from their general practitioner and were therefore not registered with a diagnosis of affective disorders in the hospital system. Therefore, it was possible that the increased risk associated with prenatal SSRIs use was partly confounded by paternal affective disorders diagnosed outside a hospital department for which we were not able to adjust. In addition, other psychiatric diseases related to SSRIs use might also contribute to the observed association.

Our study has several methodological strengths. One strength was that the linkage of several nationwide health registries in Denmark enabled us to conduct a large cohort study with virtually complete follow-up. The definition on exposure to SSRIs was based on a national registry, which eliminated the risk of recall bias caused by self-report. Another strength was that the information on ASD diagnosis was obtained independently of exposure measurement, which could also mitigate the information

For peer review only - http://bmjopen.bmj.com/site/about/guidelines.xhtml

bias. Furthermore, the availability of health registry data enabled us to adjust for a number of potential confounders including sociodemographic factors as well as parental psychiatric history. Besides, we have taken the potential effect of maternal antidepressants use during pregnancy as well as maternal mental disease into consideration. To remove the confounding factors attributing to mothers, we adjusted the maternal SSRIs use in regression model, and also restricted the analyses to children whose mothers neither received antidepressant medication during pregnancy nor had affective disorders before child birth.

Limitations need to be considered when interpreting the results of our study. Firstly, we were unable to validate actual use of SSRIs by fathers during the time period of interest because we relied on medical records of dispensed prescriptions. This may lead to misclassification of exposure status because some people may not take the medication or may take it later. Nevertheless, the misclassification was most likely non-differential, which could bias the association toward null. Besides, some patients may receive SSRIs treatment during inpatient admissions which are not included in the prescription registry. We expect this problem to be minor since those inpatients usually have severe psychiatric disorders and are more likely to continue treatment after discharge. Secondly, the 3-month cut-off point was set based on the fact that spermatogenesis takes approximately 70-90 days in humans. Whereas it may be possible that SSRIs drugs induce sperm damage at the very primitive stage. However, the results did not change markedly after extending the putative exposure period to the last 6 months prior to conception (data not shown). Thirdly, ASD in children was

For peer review only - http://bmjopen.bmj.com/site/about/guidelines.xhtml

ascertained through the DNPR and the DPCRR, which did not include those children who received the diagnosis of ASD from private psychiatrists or remained undiagnosed at the end of the follow-up. However, the prevalence of ASD in our cohort was 1.13%, which was similar to that reported in the United States during the study period (1.14%) [47]. Hence the bias introduced by case identification was expected to be minimal. Fourthly, the age of ASD diagnosis which was used as time event in Cox regression models might be affected by external and extraneous factors. If these factors are differentially distributed in exposed and unexposed group, the actual associations may be biased. We have adjusted for some factors which may influence age of diagnosis to reduce the bias to some extent. However, we could not rule out the confounding effects of unmeasured factors, which is a limitation of the study.

Conclusions

In the study, paternal SSRI use before conception was associated with an increased risk of ASD in the offspring, especially in the former users who took SSRIs over the longer term. However, null association were observed in exposed children with paternal affective disorders, and similar ASD risk were observed among exposed and unexposed siblings, which implicates that paternal underlying indications related to SSRIs use or other unmeasured confounding factors may explain the increased risk.

Footnotes

This cohort study was approved by the Danish Data Protection Agency (Document No. 2013-41-2569).

For peer review only - http://bmjopen.bmj.com/site/about/guidelines.xhtml

Contributors: HL had full access to all the data in the study and took responsibility for the integrity of the data and the accuracy of the data analysis. HL, WY and JL conceptualized and designed the study, and MHM and VE helped with its development. FY, LL and JPC conducted the statistical analysis. HL, JPC and FY interpreted the results and FY drafted the initial manuscript. All authors reviewed the manuscript and approved the final version as submitted.

Funding: The study was supported by the National Natural Science Foundation of China (81428011), the National Key Research and Development Program of China (2016YFC1000505), the European Research Council (ERC-2010-StG-260242-PROGEURO), Nordic Cancer Union (176673,186200), Danish Council for Independent Research (DFF-6110-00019), Karen Elise Jensens Fond (2016), and the Program for Clinical Research Infrastructure (PROCRIN) established by the Lundbeck Foundation and the Novo Nordisk Foundation.

Competing interests: The authors declare that they have no conflict of interest.

Statement of the independence of researchers from funders: The funding sources had no role in the design and conduct of the study; collection, management, analysis, and interpretation of the data; and preparation, review, or approval of the manuscript; and decision to submit the manuscript for publication. The researchers were independent from the funders.

Ethical approval: The study was based on secondary data. No individuals were approached as a result of the study, nor did we access any other data from the participants. This study was approved by the Danish Data Protection Agency

For peer review only - http://bmjopen.bmj.com/site/about/guidelines.xhtml

(Document No. 2013-41-2569). All procedures performed in the study involving human participants were in accordance with the ethical standards of the institutional and/or national research committee and with the 1964 Helsinki declaration and its later amendments or comparable ethical standards. Approval by an institutional review board and informed consent are not required for registry-based research in Denmark.

Data sharing statement: No additional data are available.

Reference

1. Organization WH. Depression [fact sheet no. 369]. 2012. As of September. 2015;30.
2. Alwan S, Friedman JM, Chambers C. Safety of selective serotonin reuptake inhibitors in pregnancy: a review of current evidence. CNS drugs. 2016;30(6):499-515.
3. Wen SW, Yang Q, Garner P, et al. Selective serotonin reuptake inhibitors and adverse pregnancy outcomes. American journal of obstetrics and gynecology. 2006;194(4):961-966.
4. Lund N, Pedersen LH, Henriksen TB. Selective serotonin reuptake inhibitor exposure in utero and pregnancy outcomes. Archives of pediatrics & adolescent medicine. 2009;163(10):949-954.
5. Pedersen LH, Henriksen TB, Vestergaard M, et al. Selective serotonin reuptake inhibitors in pregnancy and congenital malformations: population based cohort study. Bmj. 2009;339:b3569.
6. Oberlander TF, Reebye P, Misri S, et al. Externalizing and attentional behaviors in children of depressed mothers treated with a selective serotonin reuptake inhibitor antidepressant during pregnancy. Archives of pediatrics & adolescent medicine. 2007;161(1):22-29.
7. Nulman I, Koren G, Rovet J, et al. Neurodevelopment of children following prenatal exposure to venlafaxine, selective serotonin reuptake inhibitors, or untreated maternal depression. American Journal of Psychiatry. 2012;169(11):1165-1174.
8. Healy D, Le Noury J, Mangin D. Links between serotonin reuptake inhibition during pregnancy and neurodevelopmental delay/spectrum disorders: A systematic review of epidemiological and physiological evidence. International Journal of Risk & Safety in Medicine. 2016;28(3):125-141.
9. Clements CC, Castro VM, Blumenthal SR, et al. Prenatal antidepressant exposure is associated with risk for attention-deficit hyperactivity disorder but not autism spectrum disorder in a large health system. Molecular psychiatry. 2015;20(6):727-734.
10. Boukhris T, Sheehy O, Mottron L, et al. Antidepressant use during pregnancy and the risk of autism spectrum disorder in children. JAMA pediatrics. 2016;170(2):117-124.
11. Hadjikhani N. Serotonin, pregnancy and increased autism prevalence: is there a link? Medical Hypotheses. 2010;74(5):880-883.
12. Brown HK, Ray JG, Wilton AS, et al. Association Between Serotonergic Antidepressant Use During Pregnancy and Autism Spectrum Disorder in Children. Jama. 2017;317(15):1544-1552.
13. Sujan AC, Rickert ME, Öberg AS, et al. Associations of Maternal Antidepressant Use During the

For peer review only - http://bmjopen.bmj.com/site/about/guidelines.xhtml

First Trimester of Pregnancy With Preterm Birth, Small for Gestational Age, Autism Spectrum Disorder, and Attention-Deficit/Hyperactivity Disorder in Offspring. Jama. 2017;317(15):1553-1562.
14. Oberlander TF, Zwaigenbaum L. Disentangling Maternal Depression and Antidepressant Use During Pregnancy as Risks for Autism in Children. Jama. 2017;317(15):1533-1534.
15. Mezzacappa A, Lasica P-A, Gianfagna F, et al. Risk for Autism Spectrum Disorders According to Period of Prenatal Antidepressant Exposure: A Systematic Review and Meta-analysis. JAMA pediatrics. 2017.
16. Baird G, Simonoff E, Pickles A, et al. Prevalence of disorders of the autism spectrum in a population cohort of children in South Thames: the Special Needs and Autism Project (SNAP). The lancet. 2006;368(9531):210-215.
17. Rutter M. Incidence of autism spectrum disorders: Changes over time and their meaning*. Acta paediatrica. 2005;94(1):2-15.
18. Hill AP, Zuckerman K, Fombonne E. Epidemiology of autism spectrum disorders. Translational Approaches to Autism Spectrum Disorder: Springer 2015:13-38.
19. Newschaffer CJ, Croen LA, Daniels J, et al. The epidemiology of autism spectrum disorders*. Annu Rev Public Health. 2007;28:235-258.
20. Brown HK, Hussain-Shamsy N, Lunsky Y, et al. The association between antenatal exposure to selective serotonin reuptake inhibitors and autism: a systematic review and meta-analysis. The Journal of clinical psychiatry. 2017;78(1):e48.
21. Gidaya NB, Lee BK, Burstyn I, et al. In utero exposure to selective serotonin reuptake inhibitors and risk for autism spectrum disorder. Journal of autism and developmental disorders. 2014;44(10):2558-2567.
22. Croen LA, Grether JK, Yoshida CK, et al. Antidepressant use during pregnancy and childhood autism spectrum disorders. Archives of general psychiatry. 2011;68(11):1104-1112.
23. Rai D, Lee BK, Dalman C, et al. Parental depression, maternal antidepressant use during pregnancy, and risk of autism spectrum disorders: population based case-control study. 2013.
24. Rai D, Lee BK, Dalman C, et al. Antidepressants during pregnancy and autism in offspring: population based cohort study. Bmj. 2017;358:j2811.
25. Schirm E, Pedersen L, Tobi H, et al. Drug use among fathers around time of conception: two register based surveys from Denmark and The Netherlands. Pharmacoepidemiology and drug safety. 2004;13(9):609-613.
26. Engeland A, Bramness JG, Daltveit AK, et al. Prescription drug use among fathers and mothers before and during pregnancy. A population‐based cohort study of 106 000 pregnancies in Norway 2004‐2006. British journal of clinical pharmacology. 2008;65(5):653-660.
27. Crijns I, Bos J, Knol M, et al. Paternal drug use: before and during pregnancy. Expert opinion on drug safety. 2012;11(4):513-518.
28. Tanrikut C, Feldman AS, Altemus M, et al. Adverse effect of paroxetine on sperm. Fertility and sterility. 2010;94(3):1021-1026.
29. Safarinejad MR. Sperm DNA damage and semen quality impairment after treatment with selective serotonin reuptake inhibitors detected using semen analysis and sperm chromatin structure assay. The Journal of urology. 2008;180(5):2124-2128.
30. Lewis S, Aitken R. DNA damage to spermatozoa has impacts on fertilization and pregnancy. Cell and tissue research. 2005;322(1):33-41.
31. Pedersen CB, Gøtzsche H, Møller JØ, et al. The Danish civil registration system. Dan Med Bull.

For peer review only - http://bmjopen.bmj.com/site/about/guidelines.xhtml

2006;53(4):441-449.

32. Knudsen LB, Olsen J. The Danish Medical Birth Registry. Danish medical bulletin. 1998;45(3):320-323.

33. Kildemoes HW, Sørensen HT, Hallas J. The Danish national prescription registry. Scandinavian journal of public health. 2011;39(7 suppl):38-41.

34. Cordier S. Evidence for a role of paternal exposures in developmental toxicity. Basic & clinical pharmacology & toxicology. 2008;102(2):176-181.

35. Mors O, Perto GP, Mortensen PB. The Danish psychiatric central research register. Scandinavian journal of public health. 2011;39(7 suppl):54-57.

36. Andersen TF, Madsen M, Jørgensen J, et al. The Danish National Hospital Register. A valuable source of data for modern health sciences. Danish medical bulletin. 1999;46(3):263-268.

37. Lauritsen MB, Jørgensen M, Madsen KM, et al. Validity of childhood autism in the Danish Psychiatric Central Register: findings from a cohort sample born 1990–1999. Journal of autism and developmental disorders. 2010;40(2):139-148.

38. Christensen J, Parner ET, Vestergaard M. Antidepressant exposure in pregnancy and risk of autism spectrum disorders. Clinical epidemiology. 2013;5:449-459.

39. Hviid A, Melbye M, Pasternak B. Use of selective serotonin reuptake inhibitors during pregnancy and risk of autism. New England Journal of Medicine. 2013;369(25):2406-2415.

40. Maciag D, Simpson KL, Coppinger D, et al. Neonatal antidepressant exposure has lasting effects on behavior and serotonin circuitry. Neuropsychopharmacology. 2006;31(1):47-57.

41. Bonnin A, Levitt P. Fetal, maternal and placental sources of serotonin and new implications for developmental programming of the brain. Neuroscience. 2011;197:1.

42. Tanrikut C, Schlegel PN. Antidepressant-associated changes in semen parameters. Urology. 2007;69(1):185. e185-185. e187.

43. Elmazoudy R, Abdelhameed N, Elmasry A. Paternal dapoxetine administration induced deterioration in reproductive performance, fetal outcome, sexual behavior and biochemistry of male rats. International Journal of Impotence Research. 2015;27(6).

44. Daniels JL, Forssen U, Hultman CM, et al. Parental psychiatric disorders associated with autism spectrum disorders in the offspring. Pediatrics. 2008;121(5):e1357-e1362.

45. Larsson HJ, Eaton WW, Madsen KM, et al. Risk factors for autism: perinatal factors, parental psychiatric history, and socioeconomic status. American journal of epidemiology. 2005;161(10):916-925.

46. Jokiranta E, Brown AS, Heinimaa M, et al. Parental psychiatric disorders and autism spectrum disorders. Psychiatry research. 2013;207(3):203-211.

47. Kogan MD, Blumberg SJ, Schieve LA, et al. Prevalence of parent-reported diagnosis of autism spectrum disorder among children in the US, 2007. Pediatrics. 2009;124(5):1395-1403.

For peer review only - http://bmjopen.bmj.com/site/about/guidelines.xhtml

Table1. Baseline Characteristics of the study population

Characteristic	Paternal SSRIs use during the last 3 months prior to conception (N=6,870)	No paternal SSRIs use during the last 3 months prior to conception (N=663, 052)
**Calendar Year of birth, No. (%)**		
1998-2000	1,021(14.9)	185,784(28.0)
2001-2003	1,526(22.2)	179,952(27.1)
2004-2006	2,305(33.5)	178,664(27.0)
2007-2008	2,018(29.4)	118,652(17.9)
**Gender, No. (%)**		
Boy	3,549(51.7)	340,194(51.3)
Girl	3,321(48.3)	322,858(48.7)
**Birth Weight (g), No. (%)**		
<2500	278(4.1)	22,315(3.4)
2500-3250	1,729(25.2)	154,494(23.3)
3250-4000	3,582(52.1)	348,853(52.6)
4000-8000	1,243(18.1)	132,343(19.9)
Unknown	38(0.5)	5,047(0.8)
**Parity, No. (%)**		
1	2,693(39.2)	282,895(42.7)
2	2,433(35.4)	250,496(37.8)
≥3	1,744(25.4)	129,661(19.5)
**Preterm Birth, No. (%)**		
(<37 weeks)		
No	6,489(94.4)	630,851(95.1)
Yes	381(5.6)	32,201(4.9)
**Apgar score at 5 minutes, No. (%)**		
0-7	91(1.3)	8,224(1.2)
8-9	442(6.4)	39,941(6.0)
10	6,269(91.3)	607,634(91.7)
Unknown	68(1.0)	7,253(1.1)
**Maternal age at child birth (years), No. (%)**		
≤25	958(13.9)	99,273(15.0)
26-30	2,238(32.6)	244,419(36.9)
31-35	2,386(34.7)	225,610(34.0)
>35	1,288(18.8)	93,750(14.1)
**Paternal age at child birth (years), No. (%)**		
≤25	364(5.3)	47,490(7.2)
26-30	1,434(20.9)	180,612(27.2)
31-35	2,395(34.8)	242,702(36.6)
>35	2,677(39.0)	192,248(29.0)

For peer review only

For peer review only - http://bmjopen.bmj.com/site/about/guidelines.xhtml

Table1. Baseline Characteristics of the study population (continued)

**Maternal smoking status[a], No. (%)**		
No	4,326(63.0)	477,574(72.0)
Yes	1,334(19.4)	106,233(16.0)
Unknown	1,210(17.6)	79,245(12.0)
**Maternal AD use during pregnancy, No. (%)**		
No	6,548(94.0)	653,496(98.6)
Yes	412(6.0)	9,556(1.4)
**Maternal history of Affective disorder, No. (%)**		
No	6,607(96.2)	654,234(98.7)
Yes	263(3.8)	8,818(1.3)
**Paternal history of Affective disorder, No. (%)**		
No	6,099 (88.8)	659,639(99.5)
Yes	771(11.2)	3,413(0.5)
**Maternal history of psychiatric disorder, No. (%)**		
No	6,175(89.9)	626,840(94.5)
Yes	695(10.1)	36,212(5.5)
**Paternal history of psychiatric disorder, No. (%)**		
No	5,092 (74.1)	630,9909(95.2)
Yes	1,178(25.9)	32,062(4.8)

Abbreviations: SSRI, selective serotonin reuptake inhibitor; N, number; AD, antidepressant drugs;
[a] Maternal smoking information was missing among those children born from 2007 to 2008.

For peer review only - http://bmjopen.bmj.com/site/about/guidelines.xhtml

Table2. Association between paternal SSRIs use before conception and ASD in offspring

Paternal SSRIs use before conception	Offspring with ASD no.	Follow-up no. of person-yr	Hazard Ratio (95% CI)		
			Crude	Model 1[a]	Model 2[b]
No use during the last 3 months prior to conception	7,473	6,765,205	Ref	Ref	Ref
Use during the last 3 months prior to conception	104	61,555	1.62(1.33-1.96)	1.54(1.27-1.88)	1.43(1.18-1.74)
Sub-analysis: ***Paternal SSRIs use during the last 1 year before conception***					
No use during the last 1 year prior to conception	7,429	6,736,654	Ref	Ref	Ref
Use only from the last 1 year to the last 3 months prior to conception	71	39,422	1.71(1.35-2.16)	1.66(1.31-2.09)	1.54(1.21-1.94)
Use only during the last 3 months prior to conception	20	14,738	1.29(0.83-2.00)	1.24 (0.80-1.93)	1.17(0.75-1.82)
Use both before and during the last 3 months prior to conception	57	35,946	1.52(1.17-1.97)	1.43(1.10-1.86)	1.32(1.02-1.72)

Abbreviations: AD, antidepressant drugs; SSRI, selective serotonin reuptake inhibitor; ASD, Autism Spectrum Disorder; No., number; HR, Hazard Ratio

[a] Adjusted for calendar year of birth, sex, parity, mother age, father age, maternal smoking, mother psychiatric history, maternal AD use during pregnancy

[b] Model 1 further adjusted for father psychiatric history

For peer review only - http://bmjopen.bmj.com/site/about/guidelines.xhtml

Table3. Association between paternal SSRIs use before conception and ASD in offspring born to fathers diagnosed with affective disorder or not

Paternal SSRIs use before conception	Offspring with ASD no.	Follow-up no. of person-yr	Hazard Ratio (95% CI)		
			Crude	Model 1[a]	Model 2[b]
**Fathers with affective disorder**					
No use during the last 3 months prior to conception	58	30,908	Ref	Ref	Ref
Use during the last 3 months prior to conception	14	6,625	1.17(0.65-2.09)	1.10(0.61-1.98)	1.10(0.61-1.98)
Sub-analysis: Paternal SSRIs use during the last 1 year before conception					
No use during the last 1 year prior to conception	53	28,033	Ref	Ref	Ref
Use only from the last 1 year to the last 3 months prior to conception	8	4,082	1.07(0.51-2.25)	1.08(0.51-2.30)	1.08(0.51-2.30)
Use only during the last 3 months prior to conception	2	1,164	0.91(0.22-3.75)	0.89(0.21-3.75)	0.89(0.21-3.75)
Use both before and during the last 3 months prior to conception	9	4,254	1.16(0.57-2.35)	1.10(0.55-2.22)	1.10(0.55-2.22)
**Fathers without affective disorder**					
No use during the last 3 months prior to conception	7,415	6,752,899	Ref	Ref	Ref
Use during the last 3 months prior to conception	90	36,329	1.57(1.27-1.93)	1.52(1.23-1.87)	1.45(1.18-1.79)
Sub-analysis: Paternal SSRIs use during the last 1 year before conception					
No use during the last 1 year prior to conception	7,376	6,714,926	Ref	Ref	Ref
Use only from the last 1 year to the last 3 months prior to conception	63	37,973	1.69(1.32-2.17)	1.64(1.28-2.11)	1.57(1.23-2.02)
Use only during the last 3 months prior to conception	18	7,354	1.26(0.79-2.00)	1.23(0.77-1.95)	1.19(0.75-1.88)
Use both before and during the last 3 months prior to conception	48	28,974	1.45(1.09-1.93)	1.38(1.04-1.84)	1.32(0.99-1.76)

Abbreviations: AD, antidepressant drugs; SSRI, selective serotonin reuptake inhibitor; ASD, Autism Spectrum Disorder; No., number; HR, Hazard Ratio

[a] Adjusted for calendar year of birth, parity, mother age, father age, maternal smoking, mother psychiatric history , maternal AD use during pregnancy

[b] Model 1 further adjusted for father psychiatric history

For peer review only

Table4. Paternal SSRI use before pregnancy and ASD in exposed and unexposed siblings from 5,479 families

Paternal SSRIs use before conception	Number of offspring no.	Offspring with ASD no.	Hazard Ratio (95% CI)	
			Crude	Model 1[a]
No use during the last 3 months prior to conception	2,792	45	Ref	Ref
Use during the last 3 months prior to conception	2,687	23	0.53(0.31-0.91)	0.74(0.34-1.59)

Abbreviations: AD, antidepressant drugs; SSRI, selective serotonin reuptake inhibitor; ASD, Autism Spectrum Disorder; No., number; HR, Hazard Ratio

[a] Adjusted for sex, parity, mother age, father age, maternal smoking, maternal AD use during pregnancy

For peer review only

For peer review only - http://bmjopen.bmj.com/site/about/guidelines.xhtml

TablesS1. Association between paternal SSRIs use before conception and ASD in offspring: analyses stratified by gender

Paternal SSRIs use before conception	Offspring with ASD no.	Follow-up no. of person-yr	Hazard Ratio (95% CI)		
			Crude	Model 1[a]	Model 2[b]
**Boys**					
No use during the last 3 months prior to conception	6,005	3,459,592	Ref	Ref	Ref
Use during the last 3 months prior to conception	84	31,642	1.61(1.30-2.00)	1.55(1.25-1.92)	1.44(1.16-1.79)
Sub-analysis: ***Paternal SSRIs use during the last 1 year before conception***					
No use during the last 1 year prior to conception	5,969	3,444,956	Ref	Ref	Ref
Use only from the last 1 year to the last 3 months prior to conception	58	20,031	1.74(1.34-2.25)	1.69(1.30-2.19)	1.58(1.22-2.05)
Use only during the last 3 months prior to conception	18	7,622	1.42(0.89-2.26)	1.39(0.87-2.21)	1.31(0.83-2.09)
Use both before and during the last 3 months prior to conception	44	18,623	1.44 (1.07-1.93)	1.36(1.01-1.84)	1.26(0.94-1.71)
**Girls**					
No use during the last 3 months prior to conception	1,468	3,305,613	Ref	Ref	Ref
Use during the last 3 months prior to conception	20	29,914	1.63(1.05-2.53)	1.54(0.99-2.40)	1.41(0.90-2.20)
Sub-analysis: ***Paternal SSRIs use during the last 1 year before conception***					
No use during the last 1 year prior to conception	1,460	3,291,698	Ref	Ref	Ref
Use only from the last 1 year to the last 3 months prior to conception	13	19,390	1.61(0.93-2.78)	1.53(0.88-2.64)	1.37(0.79-2.38)
Use only during the last 3 months prior to conception	2	7,116	0.67(0.17-2.69)	0.64(0.16-2.55)	0.59(0.15-2.36)
Use both before and during the last 3 months prior to conception	13	75,323	1.84(1.06-3.17)	1.74(1.01-3.01)	1.59(0.92-2.75)

Abbreviations: AD, antidepressant drugs; SSRI, selective serotonin reuptake inhibitor; ASD, Autism Spectrum Disorder; No., number; HR, Hazard Ratio

[a] Adjusted for calendar year of birth, parity, mother age, father age, maternal smoking, mother psychiatric history, maternal AD use during pregnancy

[b] Model 1 further adjusted for father psychiatric history

For peer review only

TableS2. Association between paternal SSRIs use before conception and ASD in offspring born to mother who neither received antidepressants medication during pregnancy nor had affective disorders before birth of child

Paternal SSRIs use before conception	Offspring with ASD no.	Follow-up no. of person-yr	Hazard Ratio (95% CI)		
			Crude	Model 1[a]	Model 2[b]
No use during the last 3 months prior to conception	7,184	6,627,720	Ref	Ref	Ref
Use during the last 3 months prior to conception	91	57,081	1.55(1.26-1.91)	1.57(1.28-1.93)	1.45(1.18-1.79)
Sub-analysis:					
Paternal SSRIs use during the last 1 year before conception					
No use during the last 1 year prior to conception	7,144	6,600,852	Ref	Ref	Ref
Use only from the last 1 year to the last 3 months prior to conception	64	37,032	1.67(1.30-2.13)	1.68(1.31-2.15)	1.56(1.22-1.99)
Use only during the last 3 months prior to conception	16	13,634	1.13(0.69-1.85)	1.15(0.71-1.89)	1.08(0.66-1.77)
Use both before and during the last 3 months prior to conception	51	33,283	1.50(1.13-1.97)	1.50(1.13-1.97)	1.38(1.05-1.82)

Abbreviations: AD, antidepressant drugs; SSRI, selective serotonin reuptake inhibitor; ASD, Autism Spectrum Disorder; No., number; HR, Hazard Ratio

[a] Adjusted for calendar year of birth, parity, mother age, father age, maternal smoking, mother psychiatric history

[b] Model 1 further adjusted for father psychiatric history

For peer review only

For peer review only - http://bmjopen.bmj.com/site/about/guidelines.xhtml

STROBE Statement—checklist of items that should be included in reports of observational studies

	Item No	Recommendation	Page No.
**Title and abstract**	1	(*a*) Indicate the study's design with a commonly used term in the title or the abstract	2
		(*b*) Provide in the abstract an informative and balanced summary of what was done and what was found	2-3
**Introduction**			
Background/rationale	2	Explain the scientific background and rationale for the investigation being reported	4
Objectives	3	State specific objectives, including any prespecified hypotheses	5
**Methods**			
Study design	4	Present key elements of study design early in the paper	6
Setting	5	Describe the setting, locations, and relevant dates, including periods of recruitment, exposure, follow-up, and data collection	6-8
Participants	6	(*a*) *Cohort study*—Give the eligibility criteria, and the sources and methods of selection of participants. Describe methods of follow-up<br>*Case-control study*—Give the eligibility criteria, and the sources and methods of case ascertainment and control selection. Give the rationale for the choice of cases and controls<br>*Cross-sectional study*—Give the eligibility criteria, and the sources and methods of selection of participants	6-8
		(*b*) *Cohort study*—For matched studies, give matching criteria and number of exposed and unexposed<br>*Case-control study*—For matched studies, give matching criteria and the number of controls per case	
Variables	7	Clearly define all outcomes, exposures, predictors, potential confounders, and effect modifiers. Give diagnostic criteria, if applicable	6-8
Data sources/ measurement	8*	For each variable of interest, give sources of data and details of methods of assessment (measurement). Describe comparability of assessment methods if there is more than one group	6-8
Bias	9	Describe any efforts to address potential sources of bias	8-10
Study size	10	Explain how the study size was arrived at	10
Quantitative variables	11	Explain how quantitative variables were handled in the analyses. If applicable, describe which groupings were chosen and why	8-9
Statistical methods	12	(*a*) Describe all statistical methods, including those used to control for confounding	8-10
		(*b*) Describe any methods used to examine subgroups and interactions	9
		(*c*) Explain how missing data were addressed	9
		(*d*) *Cohort study*—If applicable, explain how loss to follow-up was addressed<br>*Case-control study*—If applicable, explain how matching of cases and controls was addressed<br>*Cross-sectional study*—If applicable, describe analytical methods taking account of sampling strategy	Not applicable
		(*e*) Describe any sensitivity analyses	9-10

For peer review only - http://bmjopen.bmj.com/site/about/guidelines.xhtml

**Results**			
Participants	13*	(a) Report numbers of individuals at each stage of study—eg numbers potentially eligible, examined for eligibility, confirmed eligible, included in the study, completing follow-up, and analysed	10
		(b) Give reasons for non-participation at each stage	Not applicable
		(c) Consider use of a flow diagram	
Descriptive data	14*	(a) Give characteristics of study participants (eg demographic, clinical, social) and information on exposures and potential confounders	10
		(b) Indicate number of participants with missing data for each variable of interest	22
		(c) *Cohort study*—Summarise follow-up time (eg, average and total amount)	11
Outcome data	15*	*Cohort study*—Report numbers of outcome events or summary measures over time	10-11
		Case-control study—Report numbers in each exposure category, or summary measures of exposure	
		Cross-sectional study—Report numbers of outcome events or summary measures	
Main results	16	(*a*) Give unadjusted estimates and, if applicable, confounder-adjusted estimates and their precision (eg, 95% confidence interval). Make clear which confounders were adjusted for and why they were included	11
		(*b*) Report category boundaries when continuous variables were categorized	21
		(*c*) If relevant, consider translating estimates of relative risk into absolute risk for a meaningful time period	Not relevant
Other analyses	17	Report other analyses done—eg analyses of subgroups and interactions, and sensitivity analyses	11-12
**Discussion**			
Key results	18	Summarise key results with reference to study objectives	12
Limitations	19	Discuss limitations of the study, taking into account sources of potential bias or imprecision. Discuss both direction and magnitude of any potential bias	15-16
Interpretation	20	Give a cautious overall interpretation of results considering objectives, limitations, multiplicity of analyses, results from similar studies, and other relevant evidence	13-14
Generalisability	21	Discuss the generalisability (external validity) of the study results	14-15
**Other information**			
Funding	22	Give the source of funding and the role of the funders for the present study and, if applicable, for the original study on which the present article is based	17

*Give information separately for cases and controls in case-control studies and, if applicable, for exposed and unexposed groups in cohort and cross-sectional studies.

Note: An Explanation and Elaboration article discusses each checklist item and gives methodological background and published examples of transparent reporting. The STROBE checklist is best used in conjunction with this article (freely available on the Web sites of PLoS Medicine at http://www.plosmedicine.org/, Annals of Internal Medicine at http://www.annals.org/, and Epidemiology at http://www.epidem.com/). Information on the STROBE Initiative is available at www.strobe-statement.org.

For peer review only

For peer review only - http://bmjopen.bmj.com/site/about/guidelines.xhtml
